# Dynamically Normalized Pupillometry for Detecting Delayed Cerebral Ischemia After Aneurysmal Subarachnoid Hemorrhage

**DOI:** 10.1097/CCE.0000000000001135

**Published:** 2024-07-31

**Authors:** Julian Klug, Joana Martins, Ignazio De Trizio, Emmanuel Carrera, Miodrag Filipovic, Isabel Charlotte Hostettler, Urs Pietsch

**Affiliations:** 1 Division of Perioperative Intensive Care Medicine, Cantonal Hospital St.Gallen, St. Gallen, Switzerland.; 2 Stroke Research Group, Department of Clinical Neurosciences, University Hospital and Faculty of Medicine, Geneva, Switzerland.; 3 Department of Neurosurgery, Cantonal Hospital St. Gallen, St. Gallen, Switzerland.; 4 Department of Emergency Medicine, Inselspital, Bern University Hospital, University of Bern, Bern, Switzerland.

**Keywords:** constriction velocity, delayed cerebral ischemia, neuromonitoring, pupillometry, subarachnoid hemorrhage

## Abstract

**OBJECTIVES:**

Delayed cerebral ischemia (DCI) is a major driver of morbidity after aneurysmal subarachnoid hemorrhage (aSAH). Quantitative pupillometry has been shown to be of prognostic value after acute neurological injury. However, the evidence for the use of pupillometric features for the detection of DCI has been conflicting. The aim of this study was to investigate the prognostic value of frequent pupillometric monitoring for DCI detection.

**DESIGN:**

Observational cohort study from a prospective aSAH registry.

**SETTING:**

Tertiary referral center.

**PATIENTS:**

Adult patients with confirmed aSAH admitted to the ICU between March 2019 and December 2023.

**INTERVENTIONS:**

None.

**MEASUREMENTS AND MAIN RESULTS:**

One hundred fourteen patients were included, of which 31 (27.2%) suffered from DCI. All patients underwent frequent pupillometry (every 3 hr). We determined the absolute value of the neurological pupil index (NPi) and constriction velocity (CV), and their value normalized to the maximal recorded value between the admission and the pupillometry measure to account for personalized baselines. The association between pupillometry values and the occurrence of DCI within 6–24 hours was investigated. Normalized CV had the best discriminative performance to identify DCI within 8 hours, with an area under the receiver operating characteristic curve of 0.82 (95% CI, 0.69–0.91). NPi, as well as non-normalized metrics, were not significantly associated with DCI.

**CONCLUSIONS:**

Normalized CV has a clinically and statistically significant association with the occurrence of DCI after aSAH. Frequent quantitative pupillometry could improve the multimodal monitoring of patients after aSAH with the goal of improving the identification of patients likely to benefit from therapeutic interventions.

**KEY POINTS**

**Question**: Can quantitative pupillometry be used to monitor for delayed cerebral ischemia (DCI) in aneurysmal subarachnoid hemorrhage (aSAH)?

**Findings**: In this cohort study, pupil constriction velocity (CV) dynamically normalized to the maximal recorded value between the admission and the pupillometry measure was predictive of DCI within 8 hours, with an area under the receiver operating characteristic curve of 0.82 (95% CI, 0.69–0.91). Neurological pupil index, as well as non-normalized metrics, were not significantly associated with DCI.

**Meaning**: Normalized CV accounting for patients’ personalized baseline, derived from frequent quantitative pupillometry is predictive of DCI in aSAH.

Aneurysmal subarachnoid hemorrhage (aSAH) is associated with significant morbidity and mortality. Although survival has improved in the past few decades (1), survivors commonly have functional limitations in daily activities, working capacity, and ultimately quality of life (2). Delayed cerebral ischemia (DCI) is one of the main causes of secondary neurological injury and long-term impairment (3). DCI is thought to be caused by multiple mechanisms such as spreading depolarizations, disruption of the blood-brain barrier, capillary thrombosis, and neuroinflammation, of which cerebral vasospasm is the most prominent and most readily recognized (4, 5). Similarly to acute ischemic stroke, DCI leads to cerebral injury in the form of ischemia but is recognized as a distinct entity. Despite multiple trials in recent years (6–8), therapeutic options for the prevention and treatment of DCI are limited to oral nimodipine therapy and maintenance of euvolemia (4, 9). This is, in part, due to the challenges in effective risk stratification and detection of ischemia before cerebral infarction occurs (9).

In recent years, there has been increasing interest in the use of automated quantitative pupillometry for the prognostication and risk stratification in patients with acute neurological injury (10). Examination of the pupillary response using a handheld light source is part of the classical neurological examination for its strong diagnostic and prognostic value (11). Automated pupillometry provides an objective, quantitative, and reproducible assessment of the pupillary light reflex and its components (12). The Outcome Prognostication of Acute Brain Injury using the Neurological Pupil Index (ORANGE) study has shown that the neurological pupil index (NPi), a proprietary scalar index ranging from 0 to 5 and the most widely used pupillometry metric, is associated with neurological outcome and mortality after cerebral injury, including patients with aSAH, in a multicenter international cohort (13). Inter-eye differences in NPi have been associated with lateralized pathology such as midline shift and malignant cerebral edema after stroke (14–17). The constriction velocity (CV) in response to a standardized light stimulus represents a quantitative measure of pupillary reactivity as assessed in the classical neurological examination. CV has been shown to correlate with clinical outcome after cardiac arrest (18, 19) as well as with space-occupying edema, midline shift, and intracranial pressure after neurovascular and traumatic neurological injury (15, 20, 21). Both CV and inter-eye differences in NPi have been used to predict the occurrence of delirium in acute care units (22, 23).

In aSAH, both CV and NPi are associated with morbidity and mortality (13, 24–26). Examining the occurrence of DCI after aSAH, two exploratory studies reported an association between low absolute NPi values, as well as altered periodicity (27, 28). Both studies were however limited by the small number of events, with a total of seven identified DCIs per study. The relationship between NPi and DCI was further investigated in a recent larger study, which, although confirming an association between NPi and clinical outcome at 3 months as well as in-hospital mortality, could not demonstrate an association with DCI (29). This study had however several limitations, including the use of a single dichotomized pupillometry parameter, few measurements (every 8 hr), and the lack of integration of inter-eye differences (29, 30). Furthermore, by using an absolute threshold, the dynamic changes of longitudinal pupil observations were not explored.

The aim of the present study was to investigate the association of NPi and CV with DCI occurrence in aSAH patients. We sought to address limitations of prior studies by using frequent, longitudinal quantitative pupillometry, analyzing continuous values, and integrating inter-eye differences. To represent dynamic change over time, we introduced normalized pupillometry, defined as the ratio between NPI or CV and the best recorded measure since admission. This allows for the detection of small changes in pupillary reactivity over time, aiming to optimally leverage this noninvasive, safe, and physiologic biomarker.

## METHODS

### Study Setting and Design

This study was designed as a retrospective cohort study based on prospectively collected data for the evaluation of the utility of quantitative pupillometry in the detection of DCI. The study was performed at the ICU of the Cantonal Hospital St. Gallen, Switzerland. The study was conducted in accordance with the Helsinki declaration and approved by the local institutional review board (“Influences in intracranial aneurysms–multicentre approach to develop a registry and biomedical resource in patients affected by intracranial aneurysms,” Ethikkommission Ostschweiz, Ethics Committee Board No. 2022-02108, Protocol Nr. EKOS 22/179, March 14, 2024). Consent was waived in accordance with Article 34 of the Swiss Federal Act on Human Research. This article adheres to the Strengthening the Reporting of Observational Studies in Epidemiology (31) and Transparent Reporting of a multivariable prediction model for Individual Prognosis Or Diagnosis statement (32) guidelines.

### Patients

**FIGURE 1.**
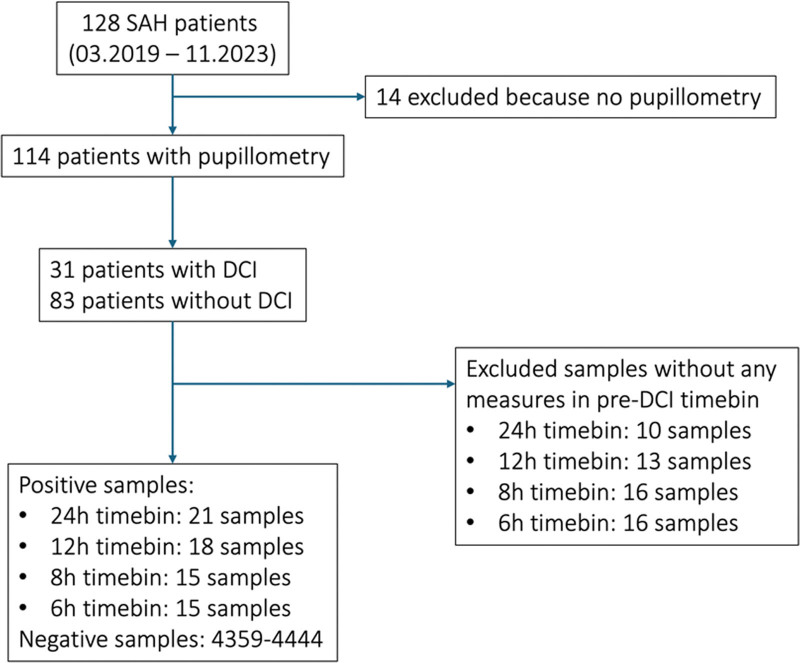
Study flow chart. DCI = delayed cerebral ischemia, SAH = subarachnoid hemorrhage.

We enrolled all consecutive adult patients (> 18 yr) with an aSAH admitted to our ICU between March 2019 and December 2023 (**Fig. 1**). Patients with nonaneurysmatic subarachnoid hemorrhage or without available pupillometry data were excluded. Patients were managed according to current guidelines (9). Aneurysm treatment modality (endovascular vs. surgical) was selected by a multidisciplinary team based on the cerebrovascular anatomy, aneurysm size, and shape in the digital subtraction angiography performed within 24 hours of admission. As part of our standard of care, all patients were admitted to the ICU for continuous monitoring and received oral nimodipine. Symptomatic acute hydrocephalus was treated with external ventricular drainage. The presence of vasospasms was investigated by routine daily transcranial Doppler. According to local protocols, all patients with aSAH were examined with quantitative pupillometry (NPi-200 Pupillometer; NeurOptics, Irvine, CA). Upon clinical deterioration or the occurrence of neurological impairment, a cerebral perfusion CT scan was obtained. Study personnel assessing outcomes were blinded to the pupillometry measurements. Treating clinicians were not blinded.

### Outcomes

DCI was defined as: 1) the occurrence of new neurological deficit (paresis, aphasia, apraxia, hemianopia, or neglect) or a decrease of at least two points on the Glasgow Coma Scale for at least 1 hour (33) or, in case patients could not be clinically assessed, suggestive alterations of neuromonitoring (decreased brain tissue oxygen pressure or near-infrared spectroscopy, altered cerebral microdialysis) and 2) evidence of focal hypoperfusion not related to a previous intervention and not attributed to other causes in a cerebral perfusion scan (29). Both criteria had to be met to qualify as DCI. We further conducted a sensitivity analysis in which the evidence of focal infarction not related to aneurysm occlusion and not attributed to other causes was required additionally to the criteria described above for the definition of DCI (29, 33). Events were recorded prospectively as part of the Swiss Study On aSAH (Swiss SOS) registry (34) and were retrospectively verified by the investigators. DCI was dated based on the first image confirming the event. As a standard of care, the clinical outcome at 1 year was prospectively assessed during an in person visit using the modified Rankin Scale (mRS). This scale, ranging from 0 (no symptoms at all) to 6 (death), is routinely used to evaluate functional outcome (35).

### Quantitative Pupillometry Features

The pupillary light reflex is quantified by quantitative pupillometry through serial measurements of pupil size recorded by an infrared camera after a calibrated light stimulation. From the obtained parameters, CV and NPi were recorded as part of clinical practice. The CV reflects the change in pupil size from baseline to minimum over time. The NPi is a proprietary scalar index ranging from 0 to 5, with greater than or equal to 3 being a normal value (12). A value is recorded for each eye for every measurement. To represent relative changes over time for a given patient, we introduce normalized quantitative pupillometry: the measured value at a given timepoint is represented as a ratio relative to the maximum observed in the same eye up to this timepoint. The reference value used for normalization changes over time and differs between eyes. Normalization was performed before the creation of timebins. Both normalized and not normalized metrics were studied. We defined normalized CV and normalized NPi as:


$$\begin{array}{l} Norm\;(CV{)}_{t}=\frac{CV_{t}+1}{\underset{0\leq it}{max}\;CV_{i}+1} \end{array}$$

$$\begin{array}{l} Norm\;(NPi{)}_{t}=\frac{NPi_{t}+1}{\underset{0\leq it}{max}\;NPi_{i}+1} \end{array}$$

where CV_t_ and NPi_t_ represent the values for a specific eye at timepoint t. If no prior measure is present, the normalized value defaults to 1.

The interpretation of normalized values is centered around 1, at which the current value is equal to the prior recorded maximum. Normalized values less than 1 represent values lower than the prior maximum and normalized values greater than 1 represent values greater than the prior maximum.

**FIGURE 2.**
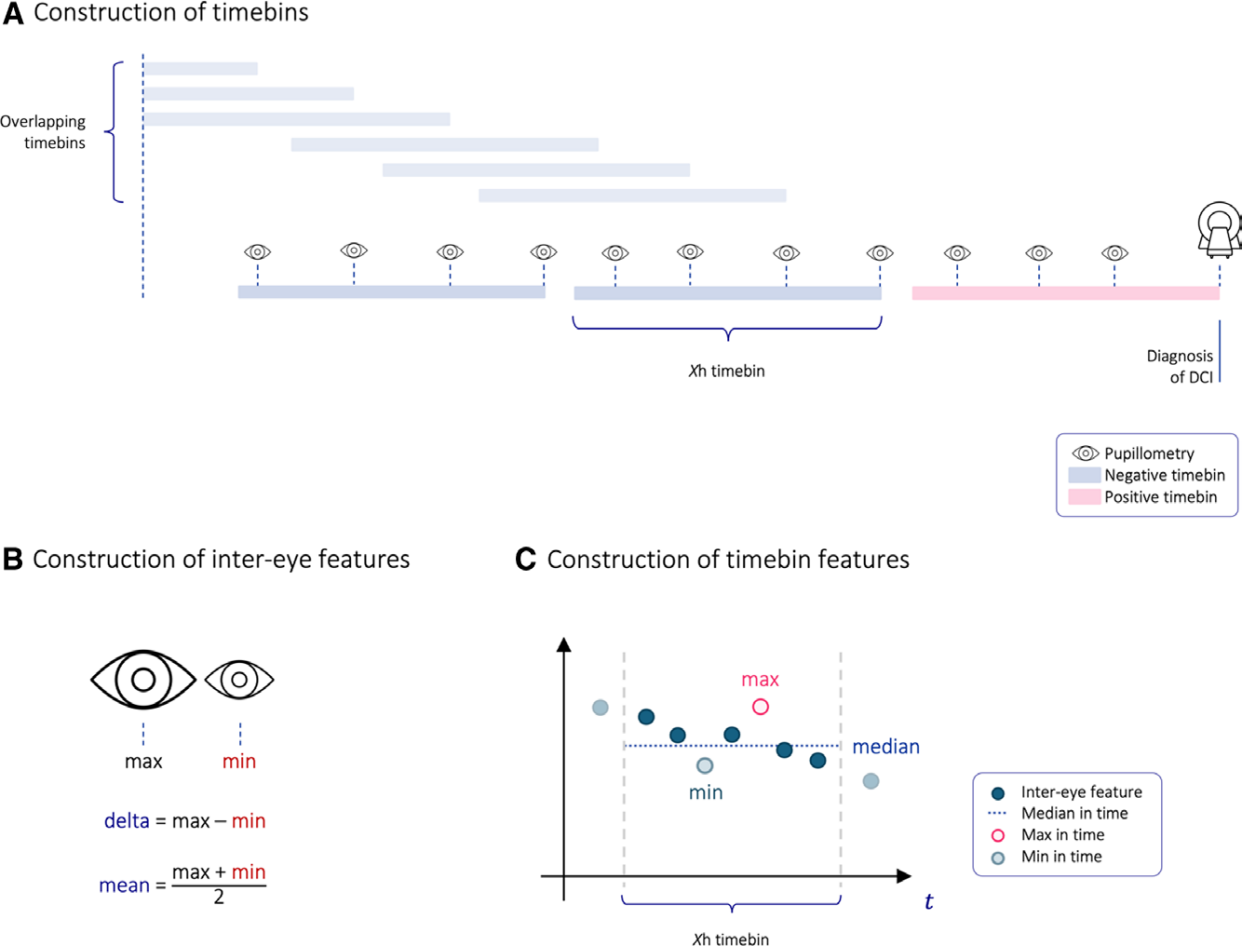
Construction of timebins and features. To find the best parameter accounting for measurements in both eyes without regard to the side of the cerebral insult while aiming to identify the best diagnostic time window before delayed cerebral ischemia (DCI), we evaluated multiple features derived from both eyes across time. **A**, An example of a time course of a patient with DCI. Binocular pupillometry was performed frequently during the patient’s ICU stay (median interval: 3 hr). As pupillometry measurements are repeated with varying intervals over time, measurements occurring close together in time were grouped into timebins. Timebins of varying sizes *X* (6, 8, 12, and 24 hr) were analyzed and contain all measurements that occurred in the *X* hours before. Positive timebins ended with DCI (*magenta*), negative samples were defined as ending with a pupillometry measure (*blue*). Negative timebins could overlap but not with a positive timebin. Within a timebin, all measures were analyzed jointly. For patients with DCI, all measures after the event were censored. **B**, To resolve parity between eyes, every binocular pupillometry measurement was summarized into inter-eye mean, minimum, maximum, and delta. **C**, For every inter-eye feature, timebins were condensed into a minimum, maximum, and median value across time. Multiple measures over time of a single inter-eye feature over time are represented as *dark-blue dots*. For every timebin, the maximum (*magenta*), minimum (*turquoise*) and median (*light-blue*) are analyzed.

As pupillometry measurements are repeated with varying intervals over time, we grouped all measurements in timebins of length *X* (**Fig. 2*A***). Four values of *X* (6, 8, 12, and 24 hr) were evaluated. For any given analysis, all timebins were of the same length. At every timepoint, a measure for both the left and right eye was recorded. To create generalizable features, we summarized every binocular measurement into inter-eye mean, minimum, maximum, and delta (**Fig. 2*B***). For every timebin and inter-eye metric, we defined a feature for the minimum, maximum, and median in time across the respective timebin (**Fig. 2*C***). For both CV and NPi, this resulted in 12 features per timebin (**Supplemental Table 1**, http://links.lww.com/CCX/B383). For example, NPi (inter-eye mean, maximum in a 12 hr timebin) corresponds to the maximum of recorded inter-eye mean NPi in a timebin of 12 hours. Examples for the computation of features and construction of timebins are detailed in **Supplemental Methods 1** and **2** (http://links.lww.com/CCX/B383).

Timebins were labeled as containing a DCI event or not. All measurements occurring after a DCI were censored. A positive timebin was defined as ending with a DCI, thus containing the *X* hours before DCI occurrence. Negative timebins were defined by the pupillometry measurement ending it and included the *X* hours prior. Negative timebins could not overlap with a timebin including a DCI (Fig. 2*A*). All measurements were included in the analysis until either discharge from the ICU or occurrence of DCI. Patients with DCI but without measurements in the timebin ending with a DCI only contributed negative timebins.

### Statistical Methods

We used a mixed-effects model with DCI as outcome, feature as continuous fixed effect and patient as random effect to identify if a feature was associated with the occurrence of DCI within the same timebin. All reported *p* values are corrected by the Benjamini and Yekutieli (36) procedure to control for false discovery rate. To evaluate a feature’s discriminative ability, we used the area under the receiver operating characteristic curve (AUC) (37). An AUC of 0.5 indicates no discriminative ability, whereas an AUC of greater than 0.7 indicates acceptable, AUC of greater than 0.8 good discriminative ability, AUC of greater than 0.9 excellent, and an AUC of 1 perfect discriminative ability (38). We split the data into derivation and test cohorts using five-fold cross-validation to avoid overfitting when applying thresholds. To obtain a binary classification boundary for continuous metrics (CV and NPi), a threshold was obtained using Youden’s index (39) on each derivation split. This threshold was then applied to the corresponding test set to compute accuracy, positive predictive value, sensitivity, specificity, and negative predictive value. The 95% CI was obtained through bootstrapping of 1000 samples with replacement. Interquartile ranges (IQRs) are used to represent variation across data. We further evaluated the association of quantitative pupillometry with long-term outcomes using an ordinal logistic regression model. The association of mRS at 1 year with the median of inter-eye minimum over the whole ICU stay of NPi and CV measurements was investigated. Univariable and multivariable analyses with adjustment for age, World Federation of Neurological Surgeons (WFNS) scale, and modified Fisher scale were performed.

## RESULTS

### Study Population

**FIGURE 3.**
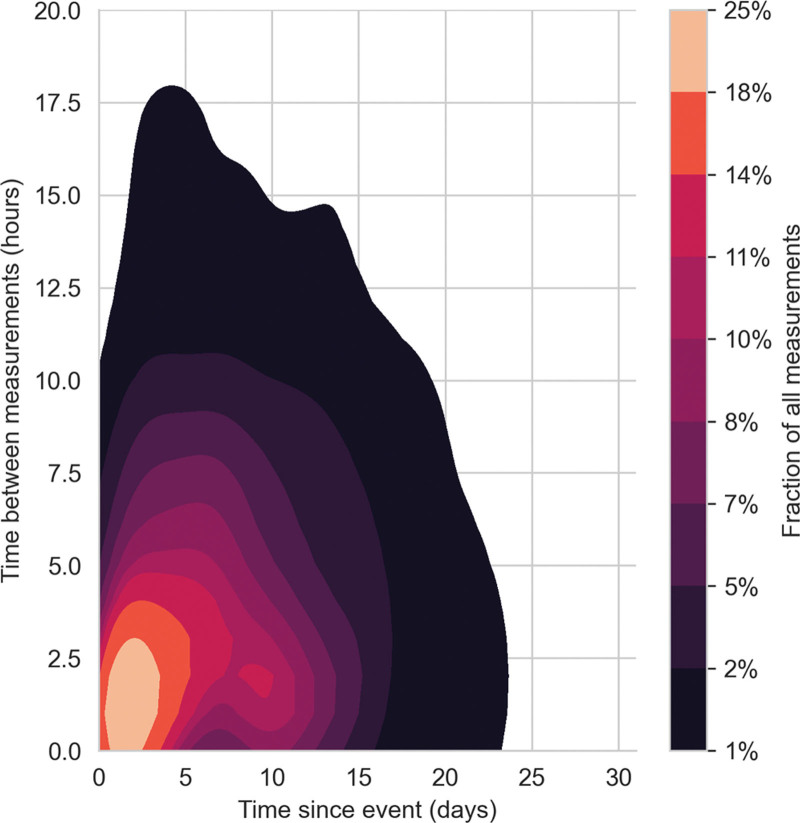
*Kernel density plot* of the interval between pupillometry measurements and the time since the subarachnoid bleed. The time between pupillometry measurements in hours is displayed on the *Y*-axis. The *X*-axis represents the time since the index event in days. The *color scale* represents the fraction of all measurements in a given region, with *warmer colors* indicating more measurements and *colder colors* less measurements.

Between March 30, 2019, and November 16, 2023, we screened 128 patients with aSAH, of whom 114 (89%) were included (Fig. 1). The median age was 58.2 years (IQR, 50.4–70.2 yr) and 77 (67.5%) were female. Upon admission, the median grade on the WFNS and modified Fisher scales were both 3 (IQR, 2–4 and IQR, 3–3, respectively) (40, 41). Most aneurysms were located in the anterior circulation with the most common locations being the anterior communicating artery (26.3%) and the middle cerebral artery (18.4%). Eighty patients (70.2%) underwent endovascular coiling, 30 (26.3%) surgical clipping, and 9 (7.8%) received no acute intervention. Twenty-six patients (83.9%) in the DCI group and 34 patients (41.0%) in the non-DCI group had evidence of vasospasm on imaging. DCI occurred in 31 patients (27.2%) at a median of 7.4 days (IQR, 4.7–10.4 d). The median ICU length of stay was 12 days (IQR, 7–16 d). Twenty-seven patients died (23.7%) during their hospital stay and median follow-up mRS was 2 (IQR, 1–6) at 1 year. Thirty-five patients (30%) were lost to follow-up after discharge. Patients with DCI had longer ICU and overall hospital stay, higher in-hospital mortality, and worse functional outcome at 1 year. Baseline characteristics are reported in **Table 1**. Quantitative pupillometry measures were recorded during the entire ICU stay. The average number of measurements per patient was 49.3 and the median interval between measures was 3.1 hours (IQR, 1.1–6.5 hr) (**Fig. 3**). The median timepoint of reference values used for normalization were recorded at 3.1 and 1.7 days for CV and NPi, respectively (**Supplemental Fig. 1**, http://links.lww.com/CCX/B383). Pupillometry measurements were split into positive and negative timebins resulting in 15–21 (0.3–0.5%) positive, and 4359–4444 negative samples, depending on the size of the timebin investigated. Ten patients (32.3%) with DCI had negative samples only, as no pupillometry was performed within the timebin in which the DCI occurred.

**TABLE 1.** Study Population Characteristics

Variable	Overall Population (*n* = 114)	DCI (*n* = 31)	No DCI (*n* = 83)
Demographics			
Age	58.2 (50.4–70.2)	56.6 (45.9–63.5)	59.8 (51.2–71.3)
Sex (female)	77 (67.5%)	19 (61.3%)	58 (69.9%)
Risk factors			
Hypertension	42 (36.8%)	11 (35.5%)	31 (37.3%)
Diabetes	3 (2.6%)	1 (3.2%)	2 (2.4%)
Aneurysm location			
Anterior communicating artery	30 (26.3%)	9 (29.0%)	21 (25.3%)
Posterior communicating artery	9 (7.9%)	2 (6.5%)	7 (8.4%)
Anterior cerebral artery	5 (4.4%)	2 (6.5%)	3 (3.6%)
Middle cerebral artery	21 (18.4%)	8 (25.8%)	13 (15.7%)
Posterior cerebral artery	0 (0.0%)	0 (0.0%)	0 (0.0%)
Internal carotid artery	7 (6.1%)	3 (9.7%)	4 (4.8%)
Vertebral/basilar artery	6 (5.3%)	0 (0.0%)	6 (7.2%)
Admission status			
Glasgow Coma Scale	13 (5–15)	13 (8–14)	13 (3–15)
World Federation of Neurological Surgeons Scale	3 (2–4)	3 (2–4)	3 (2–5)
modified Fisher Scale	3 (3–3)	3 (3–3)	3 (3–3)
Intubated	29 (25.4%)	6 (19.4%)	23 (27.7%)
Acute treatment			
Coiling	80 (70.2%)	19 (61.3%)	61 (73.5%)
Clipping	30 (26.3%)	13 (41.9%)	17 (20.5%)
Outcomes			
Vasospasm^a^	60 (52.6%)	26 (83.9%)	34 (41.0%)
ICU length of stay (d)	12 (7–16)	16 (12–20)	9 (7–15)
Hospital length of stay (d)	18 (14–25)	22 (16–29)	18 (13–22)
Hospital mortality	27 (23.7%)	10 (32.3%)	17 (20.5%)
1-yr modified Rankin Scale	2 (1–6)	3 (3–6)	2 (1–4)

DCI = delayed cerebral ischemia.

a Detected by transcranial Doppler, CT, or digital subtraction angiography.

Numbers are reported as median (interquartile ratio) and *n* (%).

### Association of Quantitative Pupillometry Features With the Occurrence of DCI

**FIGURE 4.**
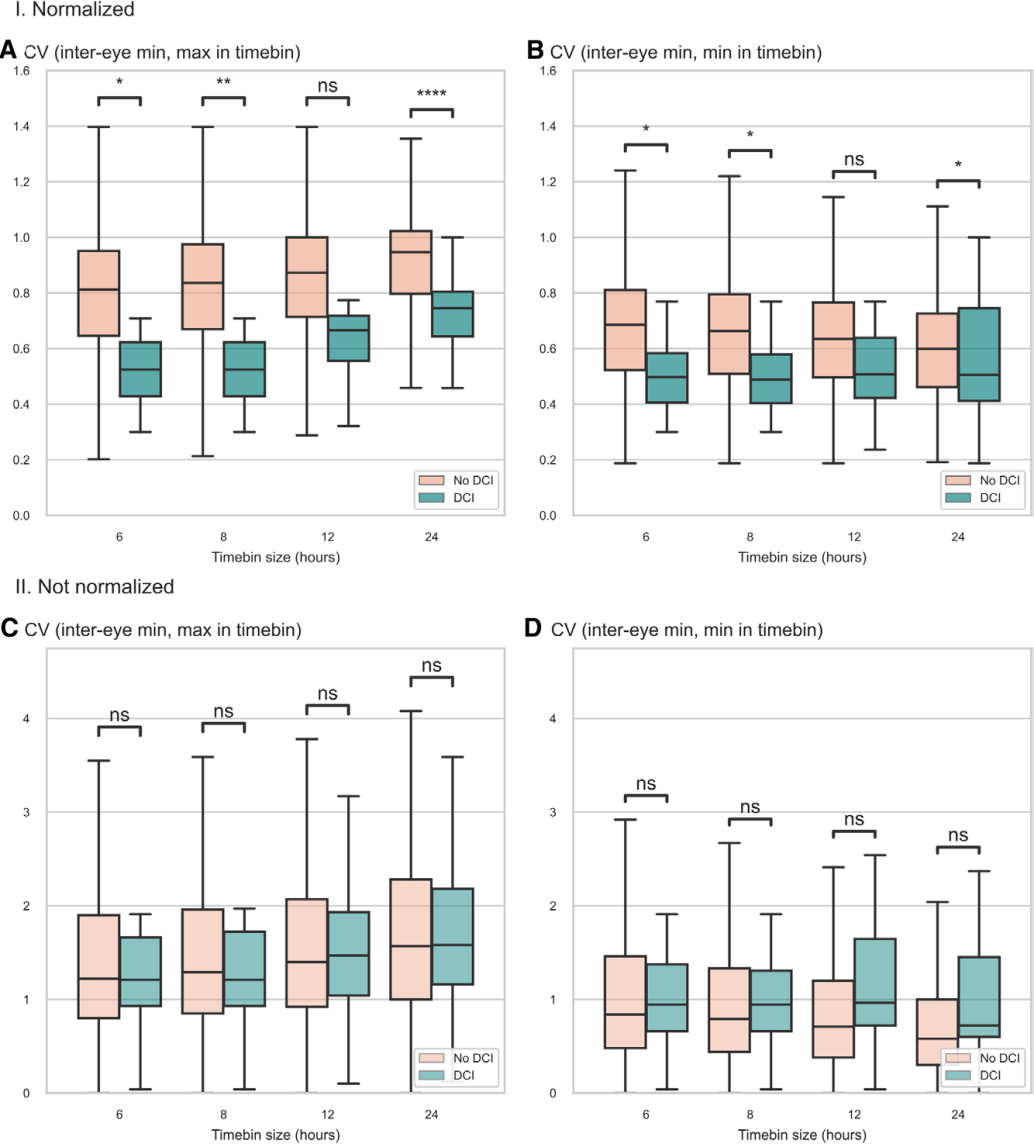
Association with the occurrence of DCI. Boxplots of selected normalized and non-normalized features across timebins according to the development of delayed cerebral ischemia (DCI). Constriction velocity (CV) (inter-eye minimum, maximum in timebin) (**A** and **C**) and CV (inter-eye minimum, minimum in timebin) (**B** and **D**) are represented as normalized (**I.**) and non-normalized features (**II.**) across all timebins. Samples with DCI are color-coded in *green*, negative samples in *salmon*. Non-normalized CV is expressed in mm/s, all other features have no units. *p < 0.05, **p < 0.01, ***p < 0.001, ****p < 0.0001. ns = not significant.

Without normalization, NPi and CV were not associated with the occurrence of DCI for all timebin lengths (**Supplemental Results 1**, **Supplemental Figs. 2** and **3**, http://links.lww.com/CCX/B383). After normalization, CV (inter-eye minimum, maximum in timebin) and CV (inter-eye minimum, minimum in timebin) showed a strong association with occurrence of DCI (*p* < 0.05) (**Fig. 4**). All other features based on normalized CV were also associated with DCI (**Supplemental Fig. 4**, http://links.lww.com/CCX/B383). Overall, normalized CV was lower before DCI with a greater inter-eye difference. Normalized NPi was not associated with DCI (*p* > 0.05) (**Supplemental Fig. 5**, http://links.lww.com/CCX/B383).

### Predictive Performance of Normalized CV Features

**FIGURE 5.**
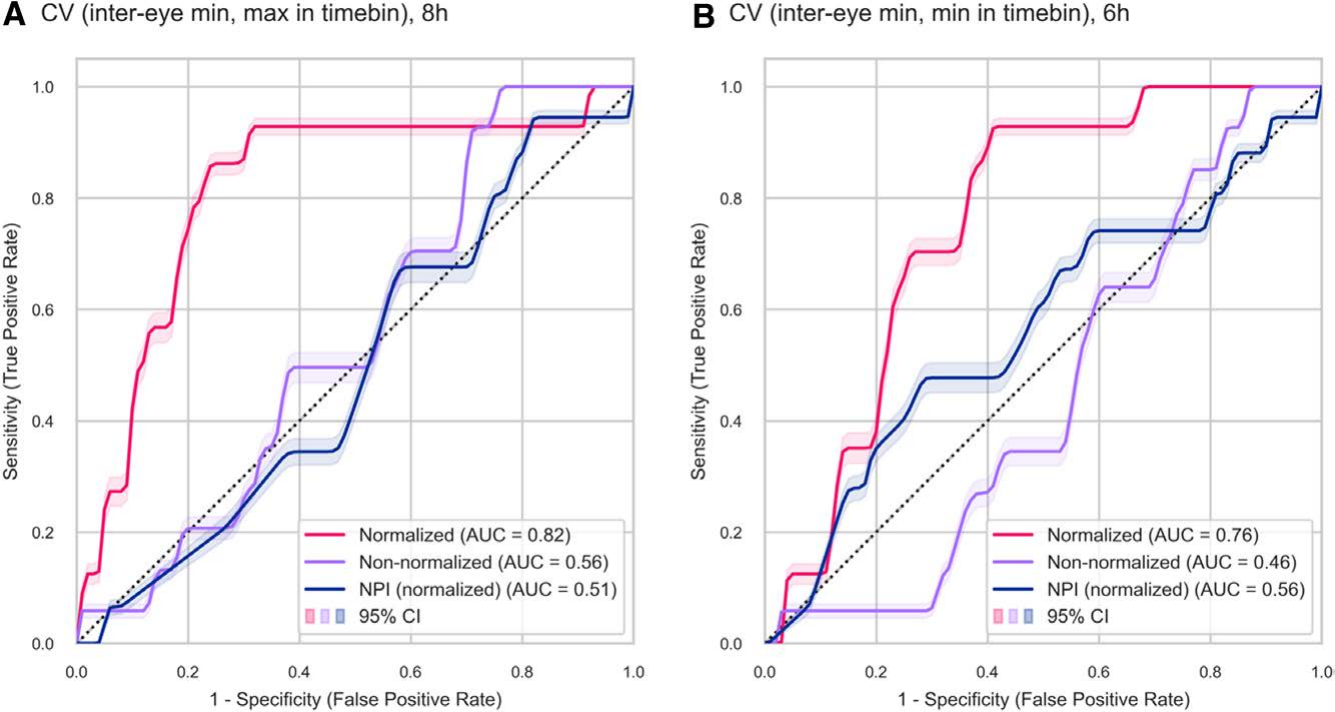
Predictive Performance. Receiver operating characteristic (ROC) curves of selected normalized and non-normalized features ROC curves for the prediction of delayed cerebral ischemia of constriction velocity (CV) (inter-eye minimum, maximum in an 8 hr timebin) (**A**) and CV (inter-eye minimum, minimum in an 6 hr timebin) (**B**), normalized (*magenta*), and non-normalized (*violet*). The corresponding normalized neurological pupil index (NPi) is shown in *blue*. AUC = area under the curve.

Normalized CV (inter-eye minimum, maximum in an 8 hr timebin) was selected based on the primary ranking metric, with an AUC of 0.82 (95% CI, 0.69–0.91) indicating good discriminative performance (**Fig. 5*A***). After five-fold cross-validation, a threshold of 0.71 was selected, resulting in a median sensitivity of 1.00 (IQR, 0.75–1.00) and median specificity of 0.73 (IQR, 0.67–0.74) in the test folds (**Supplemental Table 2**, http://links.lww.com/CCX/B383). For large prior values of maximum CV, this approximates a relative reduction of 30% in CV (**Supplemental Methods 3**, http://links.lww.com/CCX/B383). Both the corresponding non-normalized CV feature, and normalized NPi (inter-eye minimum, maximum in an 8 hr timebin) performed worse with AUCs of 0.56 (95% CI, 0.41–0.67) and 0.51 (95% CI, 0.38–0.65), respectively. We further selected normalized CV (inter-eye minimum, minimum in 6 hr timebin) for ease of bedside computation, corresponding to the worst CV measure in the last 6 hours, normalized by the best CV measured for that eye. This feature obtained an AUC of 0.76 (95% CI, 0.67–0.84) (**Fig. 5*B***). After thresholding at a median threshold of 0.63, this resulted in a median sensitivity of 1.00 (IQR, 0.75–1.00) and a median specificity of 0.61 (IQR, 0.60–0.61) in the test folds.

### Association of Quantitative Pupillometry Features With Long-Term Outcomes

In univariable analysis, both CV and NPi, expressed as median inter-eye minimum over the whole ICU stay, were associated with mRS at 1 year (*p* < 0.001 for both). After multivariable adjustment, only CV and WFNS scale were associated with mRS at 1 year (*p* = 0.003 and 0.006, respectively). NPi was not associated with long-term outcomes (*p* = 0.76) in multivariable analysis. Coefficients are reported in **Supplemental Results 2** (http://links.lww.com/CCX/B383).

### Sensitivity Analysis

All analyses were repeated using a stricter definition of DCI requiring evidence of cerebral infarction additionally to the clinical criteria. Twenty-five patients (22%) experienced DCI according to this more restrictive definition (**Supplemental Fig. 6**, http://links.lww.com/CCX/B383). The findings for this DCI definition were similar to the ones previously described: only normalized CV was associated with the occurrence of DCI (**Supplemental Figs. 7–10**, http://links.lww.com/CCX/B383). In this cohort, normalized CV (inter-eye minimum, maximum in an 8 hr timebin) and normalized CV (inter-eye minimum, minimum in 6 hr timebin) had an AUCs of 0.85 (95% CI, 0.78–0.92) and 0.80 (95% CI, 0.67–0.89), respectively.

## DISCUSSION

We conducted a large single-center study evaluating frequent quantitative pupillometry measurements and its association with DCI in patients admitted to the ICU after aSAH in line with the protocol of the recently published ORANGE study (13). Measures were grouped into timebins to study their association with the occurrence of DCI, reflecting the clinical question: Is my patient currently at risk of DCI?

We did not find an association between non-normalized pupillometric features and the occurrence of DCI, confirming the results reported by Gouvêa Bogossian et al (29). We address the main limitations identified in this study by using frequent measurements (every 3 hr) and analyzing multiple continuous pupillometric features (30). To include inter-eye difference in a disease state with heterogeneous laterality, we have analyzed the minimum, maximum, and delta between both eyes.

DCI represents a deterioration of an already altered and very heterogeneous neurological state in patients after aSAH. This dynamic change cannot be adequately captured using a single snapshot in time. To express every measurement in relation to the personalized baseline of every patient we introduce a normalized version of NPi and CV, defined relative to the best ever value recorded for the same patient in the same eye. The normalized features take into account the individual time course and the heterogeneous nature of neurological injuries after aSAH. In this exploratory analysis, we show that a decrease in normalized CV, but not normalized NPi, is associated with the occurrence of DCI. The best performing feature, the maximum value in 8 hours of the inter-eye minimum normalized CV had a very good discriminative performance with an AUC of 0.82. To simplify bedside application, we further evaluated the performance of the minimum normalized CV in a 6 hours timebin, corresponding to the worst recorded CV in the last 6 hours, divided by the best CV ever recorded for the same eye. The simplified feature maintained a good discriminative performance with an AUC of 0.76. Both features had excellent sensitivity and acceptable specificity. Nonetheless, DCI remains a rare event over the clinical time course after aSAH, resulting in a low positive predictive value for every single test (42). We thus advocate for the use of multimodal monitoring, which normalized CV could improve, especially in sedated or intubated patients who cannot be clinically assessed and in atypical neurological presentations (43, 44). We did not find an association of normalized NPi with the occurrence of DCI. When investigating the relation of NPi and CV with long-term outcomes, only CV was statically associated with 1-year mRS after multivariable adjustment. Although prior work had shown an independent association of NPi with outcomes, none was adjusted for CV (13, 24–26). NPi reflects mainly brainstem dysfunction and is designed to be only minimally affected by sedation. A prior study in patients with cortical stroke suggests that NPi may not be sensitive enough to detect cortical injury, which is the main affected area in DCI (45–47). Multiple pathways of the autonomic nervous system, integrating at the midbrain, and projecting to multiple levels of gray matter (locus coeruleus, colliculi, and cingulate cortex) are involved in the potential relationship between cortical activity and the pupillary light reflex (48). Reduced pupillary CV correlates with both cortical dysfunctions in the form of severe encephalopathy or an unreactive electroencephalogram (49), autonomic dysregulation (50–52), and a depth of sedation (53, 54). Although we cannot infer pathophysiologic conclusions from our data, the relationship between a decreased CV relatively to the patient’s baseline and DCI might involve direct cortical injury, an autonomic response associated with ischemia or vasospasm (4, 55), or the use of sedative agents in response to secondary agitation (56).

The current definition of DCI predates the era of perfusion imaging, which has brought radical changes to the care of acute ischemic stroke and has been integrated into the outcome definition in recent studies (29, 33, 57). We believe in the importance of detecting ischemia before infarction occurs to be able to prevent further cerebral injury. A sensitivity analysis requiring the presence of infarction on imaging for the definition of DCI was nonetheless included, showing similar results.

Our study has several limitations. Our analysis is based on a single center with a limited sample size. Although frequent pupillometry is part the standard monitoring in our center, 14 patients had to be excluded because no pupillometry data was recorded and 10 patients with DCI contributed only negative timebins, as no observations were available in the time span before DCI occurrence. We aimed to reduce the risk of overfitting. However, risk of type I error remains, and we strongly encourage the external validation of our results. During the study, clinicians were not blinded to pupillometry readings, which could have influenced the results. The cohort studied in this work mainly presented with aneurysms of the anterior circulation, which may affect the generalizability of the findings. The effect of sedation on the prognostic utility of pupillometry was not evaluated. Further quantitative pupillometric measures such as constriction percentage, dilation velocity, or latency were not recorded, and no conclusion can be drawn regarding their association with DCI from our findings. The spatial resolution of used pupillometers is limited to 0.03 mm and smaller differences could not be evaluated. Furthermore, the computation of normalized CV can be complicated in clinical practice, requires a record of previous measurements and is not strictly equivalent to a simple ratio. We believe that the concept of a relative decline with regards to the patient’s baseline is nonetheless useful and that centers making use of quantitative pupillometry mostly work with patient data management systems, which can be used to automatize computations. Finally, the impact of our study is limited to settings where quantitative pupillometry is available, although smartphone-based alternatives might be a low-resource option (58).

## CONCLUSIONS

We have demonstrated that normalized CV is associated with DCI after aSAH and could be an addition to the currently used multimodal monitoring. Adequate risk stratification and early detection of reversible ischemia before infarction occurs could provide us with a therapeutic window for future interventional studies. We nonetheless emphasize the importance of external validation of our results before their clinical application.

## Supplementary Material


