# Supplementary material for: Dynamically Normalized Pupillometry for Detecting Delayed Cerebral Ischemia After Aneurysmal Subarachnoid Hemorrhage
Source: Crit Care Explor. 2024 Jul 31;6(8):e1135. doi: 10.1097/CCE.0000000000001135 (PMC12333836; doi:10.1097/CCE.0000000000001135)
Supplement: Supplementary file 1 [file cc9-6-e1135-s001.pdf]

Supplementary Material to:

Dynamically Normalised Pupillometry for Detecting Delayed Cerebral Ischemia after Aneurysmal Subarachnoid Haemorrhage

Julian Klug[1, 2], Joana Martins[1], Ignazio De Trizio[1], Emmanuel Carrera[2], Miodrag Filipovic[1], Isabel Charlotte Hostettler[3], Urs Pietsch[1,4]

1. Division of Perioperative Intensive Care Medicine, Cantonal Hospital St.Gallen, St. Gallen, Switzerland.
2. Stroke Research Group, Department of Clinical Neurosciences, University Hospital and Faculty of Medicine, Geneva, Switzerland
3. Department of Neurosurgery, Cantonal Hospital St. Gallen, St. Gallen, Switzerland
4. Department of Emergency Medicine, Inselspital, Bern University Hospital, University of Bern, Bern, Switzerland

Table of Contents

Supplemental Methods 1: Computation of pupillometry features for an example patient. 3

Supplemental Methods 2: Examples of timebin construction. 4

Supplemental Table 1: List of all features. 5

Supplemental Figure 1: Distribution of reference values used for normalisation. 6

Supplemental Figure 2: Boxplots of non-normalized CV across timebins according to the development of DCI. 7

Supplemental Figure 3: Boxplots of non-normalized NPi across timebins according to the development of DCI. 8

Supplemental Figure 4: Boxplots of normalized CV across timebins according to the development of DCI. 9

Supplemental Figure 5: Boxplots of normalized NPi across timebins according to the development of DCI. 10

Supplemental Table 2: Performance of normalized automated pupillometry for the prediction of DCI. 11

Supplemental Results 1: Performance of inter-eye delta NPi for the prediction of DCI. 18

Supplemental Results 2: Coefficients for ordinal logistic regression analysis. 19

Supplemental Figure 6: Sensitivity analysis: study flow chart. 20

Supplemental Figure 7: Sensitivity analysis: Boxplots of non-normalized CV across timebins according to the development of DCI with infarction. 21

Supplemental Figure 8: Sensitivity analysis: Boxplots of non-normalized NPi across timebins according to the development of DCI with infarction. 22

Supplemental Figure 9: Sensitivity analysis: Boxplots of normalized CV across timebins according to the development of DCI with infarction. 23

Supplemental Figure 10: Sensitivity analysis: Boxplots of normalized NPi across timebins according to the development of DCI with infarction. 24

Supplemental Methods 3: Normalized threshold as a function of relative ratio. 25

References 26

Supplemental Methods 1.
Computation of pupillometry features for an example patient.

Given a patient with no occurrence of DCI, and the following raw binocular constriction velocity (CV) recordings:

**Timestamp**	**01:00**	**03:50**	**07:15**	**10:00**	**14:00**
**Left CV**	1.5	1.4	1.9	1.7	1.4
**Right CV**	1.8	1.7	2.0	0.9	1.0

This would result in normalized values of:

**Timestamp**	**01:00**	**03:50**	**07:15**	**10:00**	**14:00**
**Norm(Left CV)**	1.00	0.96	1.16	0.93	0.82
**Norm(Right CV)**	1.00	0.96	1.15	0.63	0.66

To explicit the computation of the normalized left CV at timestamp 10:00:

$$Norm(Left\ CV)_{10:00} = \frac{Left\ CV_{10:00} + 1}{\max\limits_{0 \leq i < 10:00} Left\ CV_i + 1} = \frac{1.7 + 1}{1.9 + 1} = 0.93$$

The resulting normalized inter-eye normalized CV features would be:

**Timestamp**	**01:00**	**03:50**	**07:15**	**10:00**	**14:00**
**CV, inter-eye min**	1.00	0.96	1.15	0.63	0.66
**CV, inter-eye max**	1.00	0.96	1.16	0.93	0.82
**CV, inter-eye mean**	1.00	0.96	1.16	0.78	0.74
**CV, inter-eye delta**	0.00	0.00	0.01	0.30	0.16

For a timebin size of 6h this would result in 12 normalized CV features over 5 negative timebins:

**Timebin [bounds]**	**1** [00:00-01:00]	**2** [00:00-03:50]	**3** [01:15-07:15]	**4** [04:00-10:00]	**5** [08:00-14:00]
**CV, inter-eye min, min in timebin**	1.00	0.96	0.96	0.63	0.63
**CV, inter-eye max, min in timebin**	1.00	0.96	0.96	0.93	0.82
**CV, inter-eye mean, min in timebin**	1.00	0.96	0.96	0.78	0.74
**CV, inter-eye delta, min in timebin**	0.00	0.00	0.00	0.01	0.16
**CV, inter-eye min, max in timebin**	1.00	1.00	1.15	1.15	0.66
**CV, inter-eye max, max in timebin**	1.00	1.00	1.16	1.16	0.93
**CV, inter-eye mean, max in timebin**	1.00	1.00	1.16	1.16	0.78
**CV, inter-eye delta, max in timebin**	0.00	0.00	0.01	0.30	0.30
**CV, inter-eye min, median in timebin**	1.00	0.98	1.06	0.89	0.65
**CV, inter-eye max, median in timebin**	1.00	0.98	1.06	1.05	0.88
**CV, inter-eye mean, median in timebin**	1.00	0.98	1.06	0.97	0.76
**CV, inter-eye delta, median in timebin**	0.00	0.00	0.01	0.16	0.23

Supplemental Methods 2.

Examples of timebin construction.

Given two patients (A & B) with the following fictional ICU stay:

Patient	Event	Pupillometry Timestamps						Event
A	Admission (00:00)	01:00	03:50	07:15	10:00	14:00	16:30	Discharge (17:00)
B	Admission (00:00)	01:00	03:50	07:15	10:00	14:00	16:30	DCI (17:00)

For a timebin size of 6h, this results in the following timebins:

Variable	Timeline					
**Patient A**						
**Timebin [bounds]**	**1** [00:00-01:00]	**2** [00:00-03:50]	**3** [01:15-07:15]	**4** [04:00-10:00]	**5** [08:00-14:00]	**6** [10:30-16:30]
**Number of samples**	1	2	2	2	2	2
**Labels**	Negative	Negative	Negative	Negative	Negative	Negative
**Patient B**						
**Timebin [bounds]**	**1** [00:00-01:00]	**2** [00:00-03:50]	**3** [01:15-07:15]	**4** [04:00-10:00]	**5** [11:00-17:00]	/
**Number of samples**	1	2	2	2	2	/
**Labels**	Negative	Negative	Negative	Negative	Positive	/

ICU stays were shortened to a single day for simplicity.

Supplemental Table 1.

List of all features.

Feature	Formula	Explanation
Inter-eye min, min in timebin	$\min_{t \in T}(min(m_{Lt}; m_{Rt}))$	Minimum in timebin of minimum value between both eyes at every measurement
Inter-eye max, min in timebin	$\min_{t \in T}(max(m_{Lt}; m_{Rt}))$	Minimum in timebin of maximum value between both eyes at every measurement
Inter-eye mean, min in timebin	$\min_{t \in T}(mean(m_{Lt}; m_{Rt}))$	Minimum in timebin of mean value between both eyes at every measurement
Inter-eye delta, min in timebin	$\min_{t \in T}(\| m_{Lt} - m_{Rt}\|)$	Minimum in timebin of difference between both eyes at every measurement
Inter-eye min, max in timebin	$\max_{t \in T}(min(m_{Lt}; m_{Rt}))$	Maximum in timebin of minimum value between both eyes at every measurement
Inter-eye max, max in timebin	$\max_{t \in T}(max(m_{Lt}; m_{Rt}))$	Maximum in timebin of maximum value between both eyes at every measurement
Inter-eye mean, max in timebin	$\max_{t \in T}(mean(m_{Lt}; m_{Rt}))$	Maximum in timebin of mean value between both eyes at every measurement
Inter-eye delta, max in timebin	$\max_{t \in T}(\| m_{Lt} - m_{Rt}\|)$	Maximum in timebin of difference between both eyes at every measurement
Inter-eye min, median in timebin	$\text{median}_{t \in T}(min(m_{Lt}; m_{Rt}))$	Median in timebin of minimum value between both eyes at every measurement
Inter-eye max, median in timebin	$\text{median}_{t \in T}(max(m_{Lt}; m_{Rt}))$	Median in timebin of maximum value between both eyes at every measurement
Inter-eye mean, median in timebin	$\text{median}_{t \in T}(mean(m_{Lt}; m_{Rt}))$	Median in timebin of mean value between both eyes at every measurement
Inter-eye delta, median in timebin	$\text{median}_{t \in T}(\| m_{Lt} - m_{Rt}\|)$	Median in timebin of difference between both eyes at every measurement

<u>*Legend*</u>: List of all features along with mathematical formulation and explanation. m_{Lt} and m_{Rt} are the left and right measurements at timepoint t in every timebin T. m represents a given type of measurement of CV, NPi, normalized CV and normalized NPi. NPi: Neurological pupil index; CV: constriction velocity.

Supplemental Figure 1.

Distribution of reference values used for normalisation.

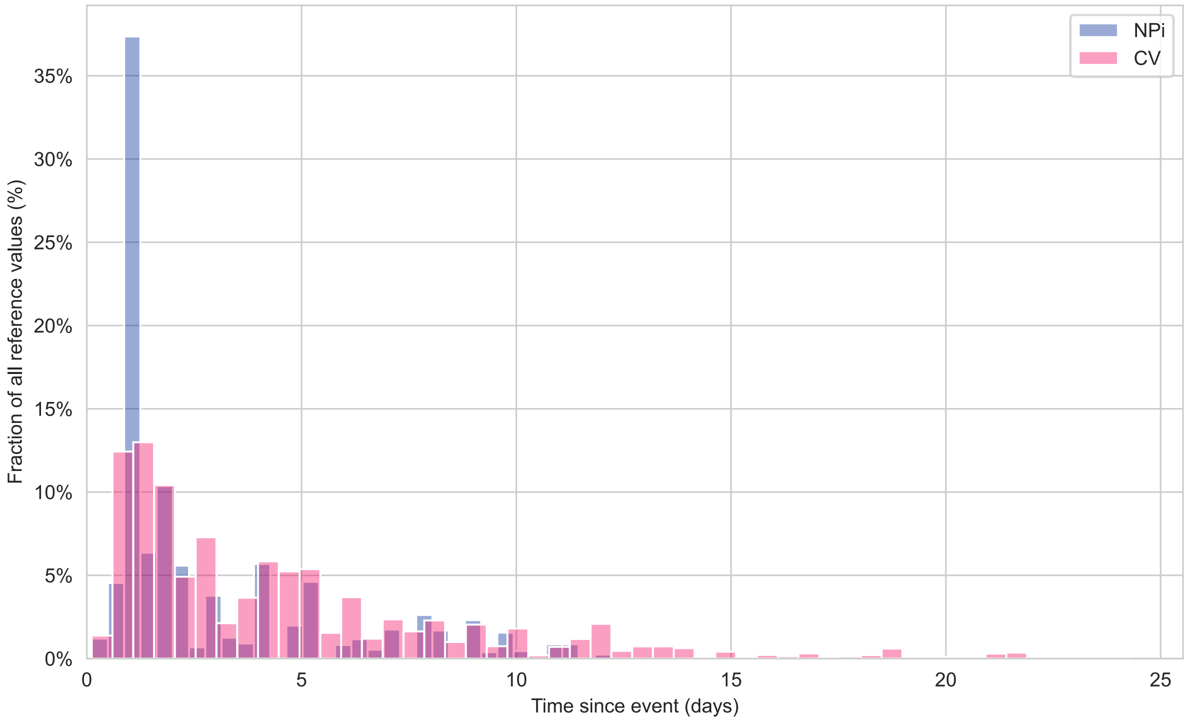

Legend: Histogram of reference values used for normalisation of pupillometry values over the ICU stay. Reference values were defined as prior maximum in the same eye for every timepoint. Values for both eyes are represented. Reference values for NPi are color-coded as blue and as magenta for CV. NPi: Neurological pupil index; CV: constriction velocity; ICU: intensive care unit.

Supplemental Figure 2.

Boxplots of non-normalized CV across timebins according to the development of DCI.

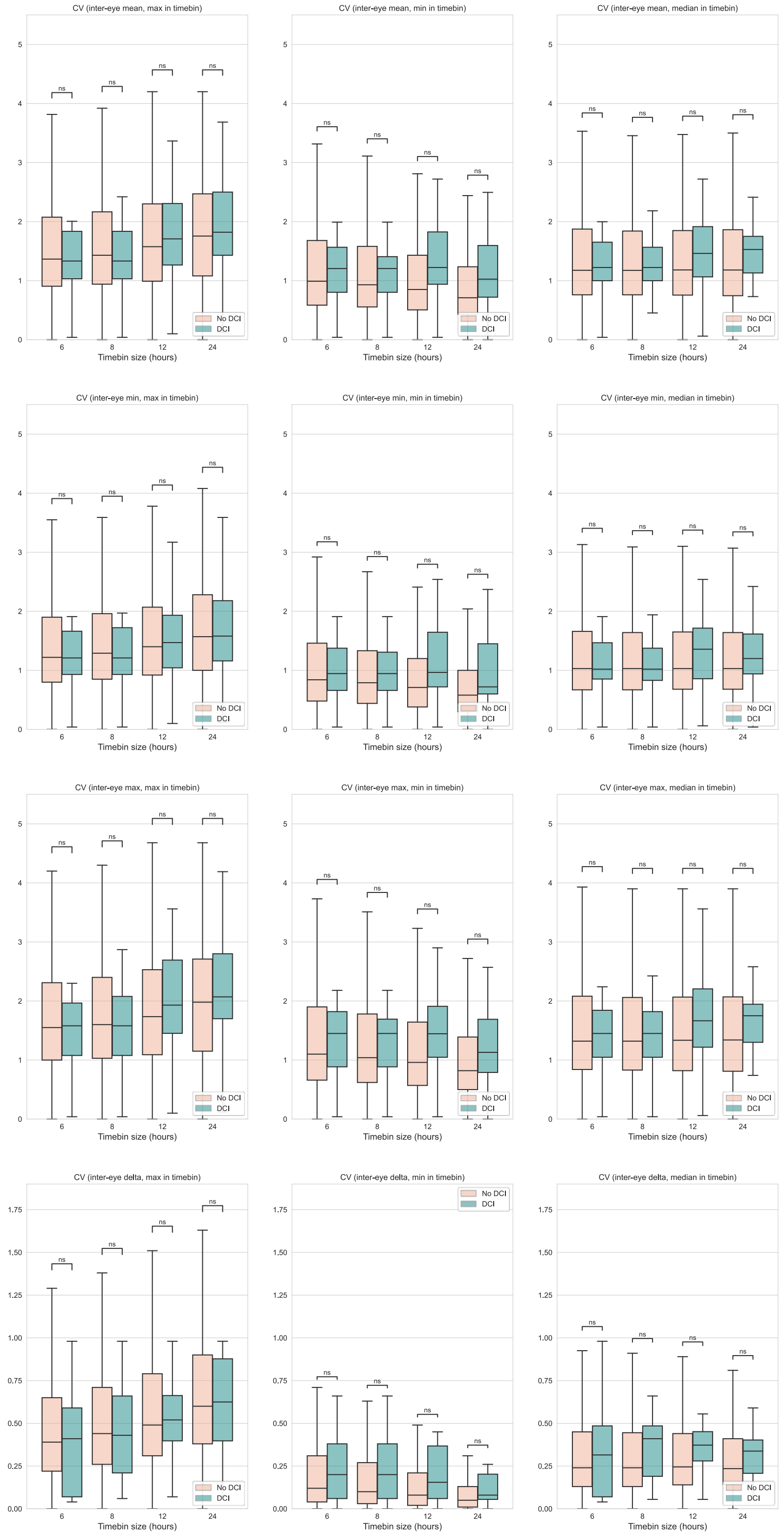

Legend: Samples with DCI are color-coded in green, negative samples in salmon. Non-normalised CV is expressed in $mm.s^{-1}$. CV: constriction velocity; DCI: delayed cerebral ischemia; ns: non-significant; *: p-value < 0.05, **: p-value < 0.01; ***: p-value < 0.001; ****: p-value < 0.0001.

Supplemental Figure 3.

Boxplots of non-normalized NPi across timebins according to the development of DCI.

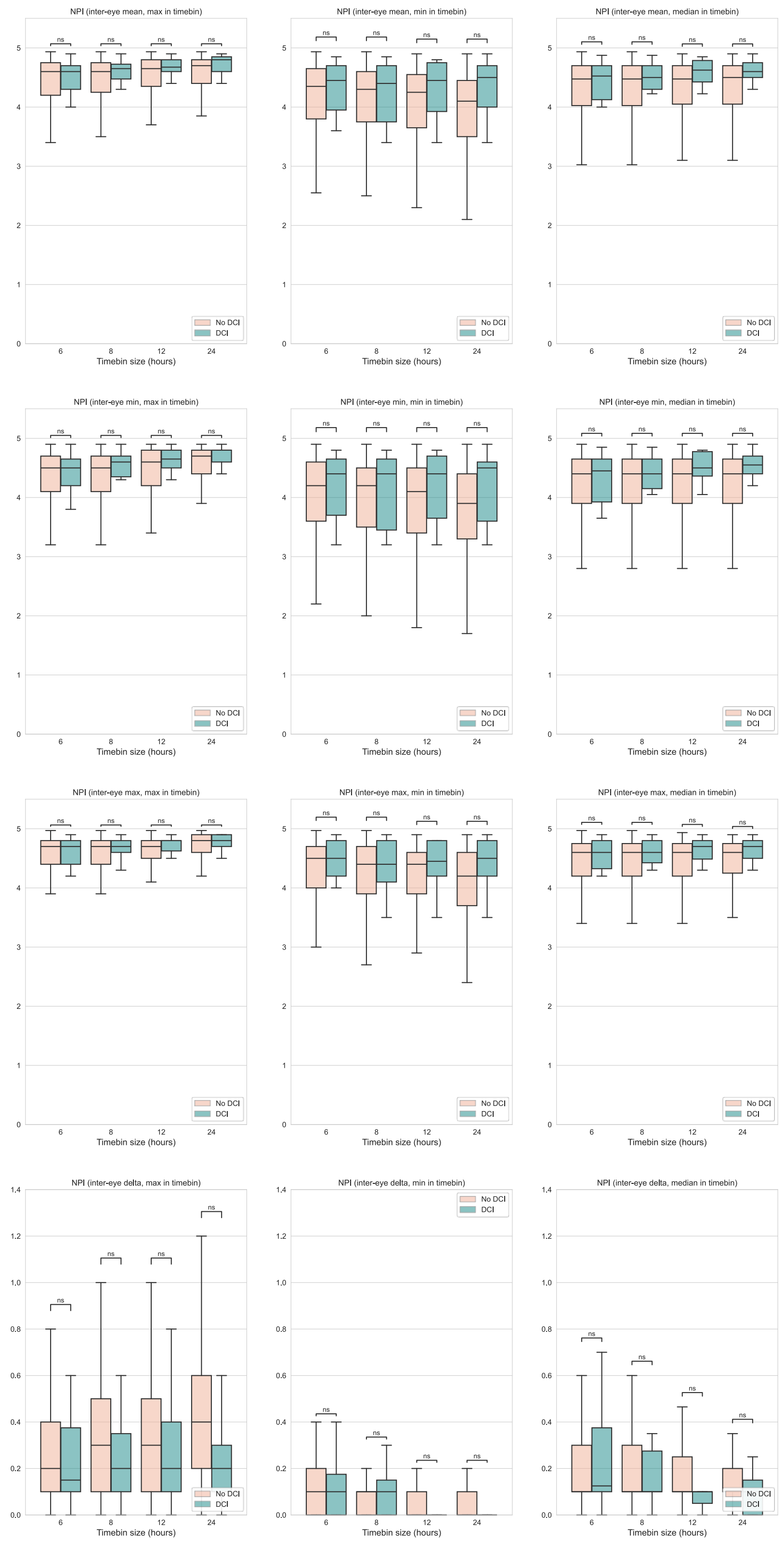

Legend: Samples with DCI are color-coded in green, negative samples in salmon. NPi: Neurological pupil index; DCI: delayed cerebral ischemia; ns: non-significant; *: p-value < 0.05, **: p-value < 0.01; ***: p-value < 0.001; ****: p-value < 0.0001.

Supplemental Figure 4.

Boxplots of normalized CV across timebins according to the development of DCI.

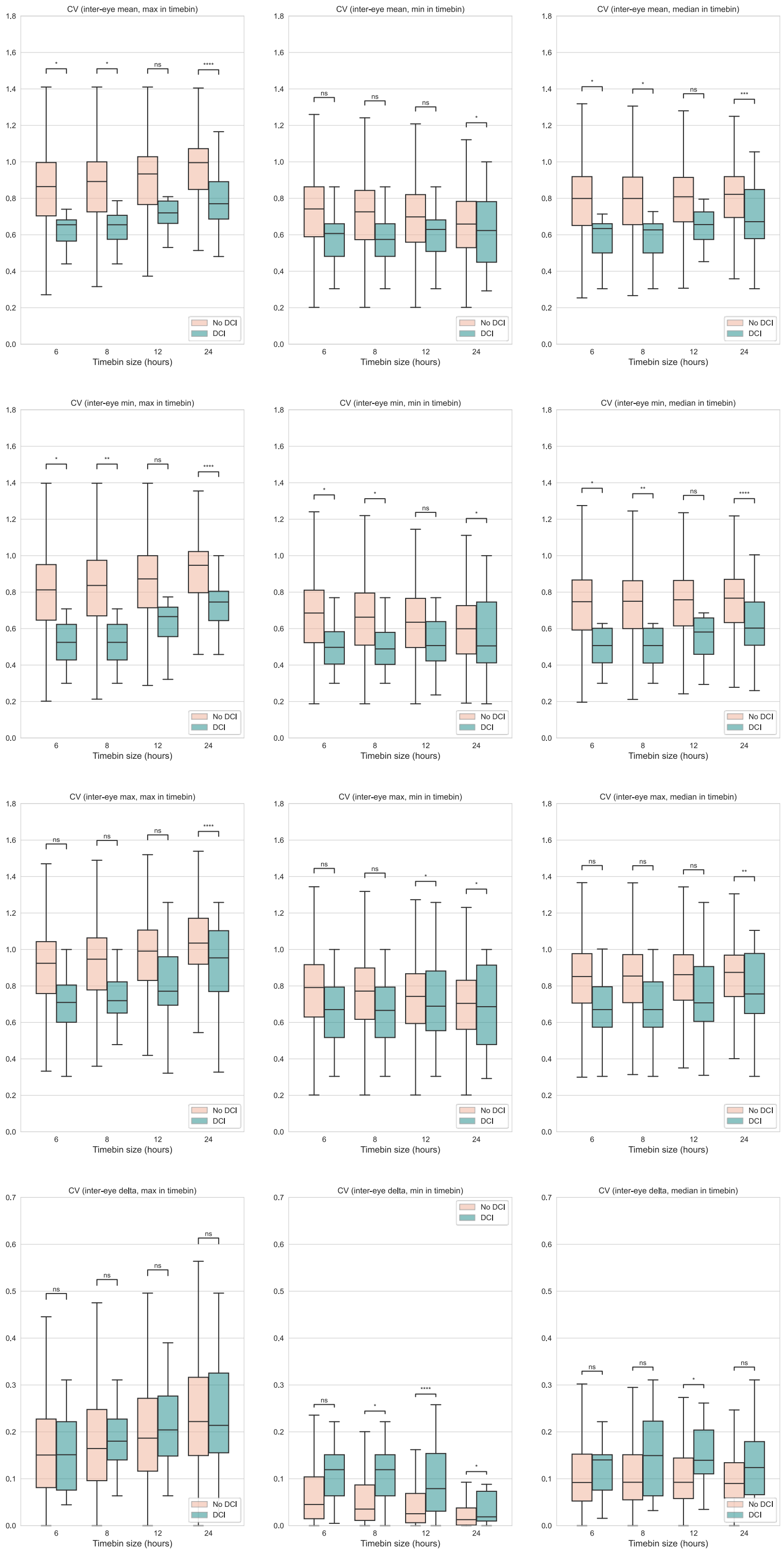

Legend: Samples with DCI are color-coded in green, negative samples in salmon. CV: constriction velocity; DCI: delayed cerebral ischemia; ns: non-significant; *: p-value < 0.05, **: p-value < 0.01; ***: p-value < 0.001; ****: p-value < 0.0001.

Supplemental Figure 5.

Boxplots of normalized NPi across timebins according to the development of DCI.

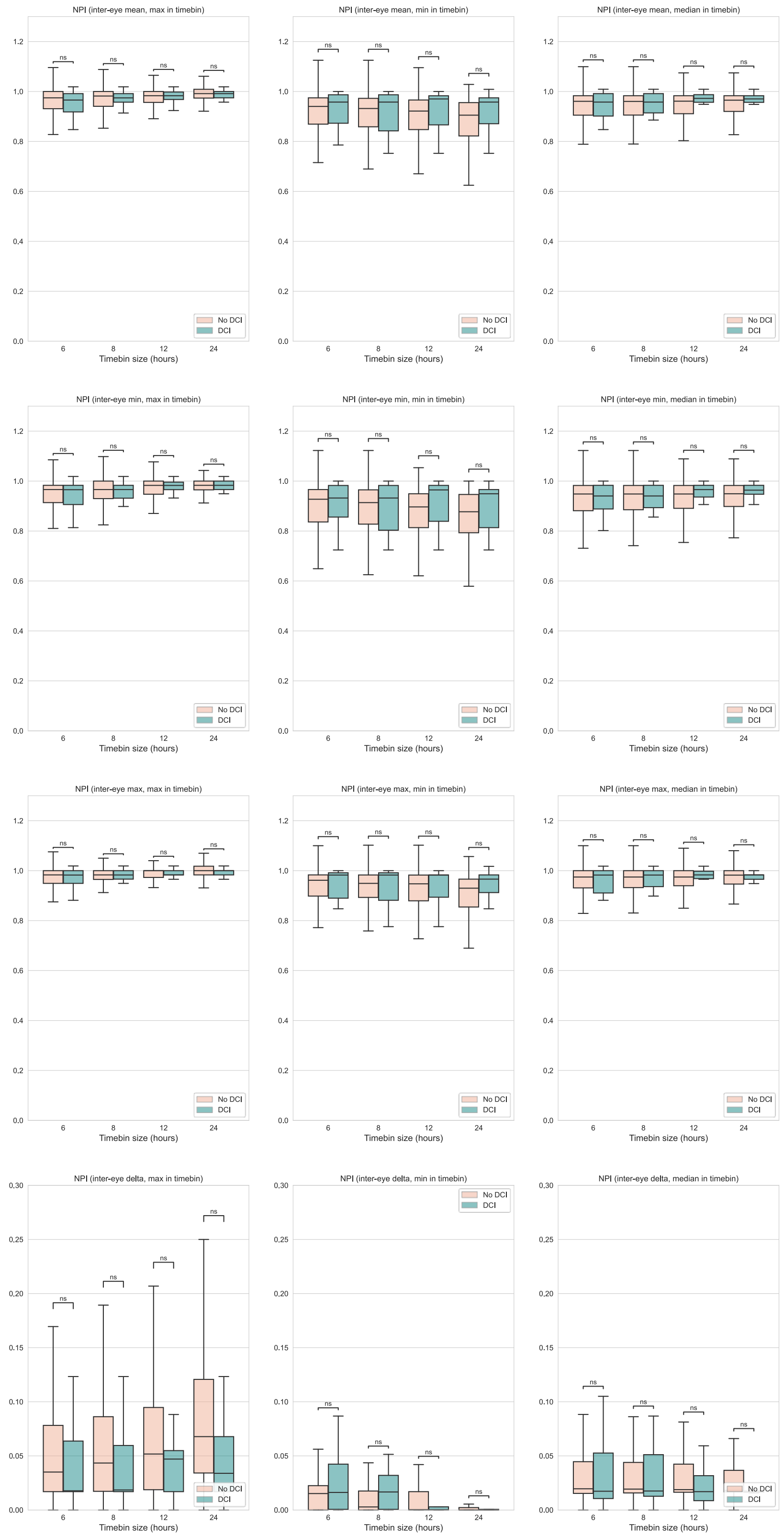

Legend: Samples with DCI are color-coded in green, negative samples in salmon. NPi: Neurological pupil index; DCI: delayed cerebral ischemia; ns: non-significant; *: p-value < 0.05, **: p-value < 0.01; ***: p-value < 0.001; ****: p-value < 0.0001.

Supplemental Table 2.

Performance of normalized automated pupillometry for the prediction of DCI.

Feature	Accuracy	Precision (PPV)	Recall (Sensitivity)	Specificity	NPV	ROC AUC
**6h timebin**						
NPI (inter-eye mean, max in timebin)	0.59 (0.58-0.59)	0.01 (0.00-0.01)	0.40 (0.00-0.60)	0.58 (0.58-0.59)	1.00 (1.00-1.00)	0.55 (95% CI 0.43-0.70)
NPI (inter-eye mean, min in timebin)	0.60 (0.59-0.76)	0.00 (0.00-0.01)	0.20 (0.00-0.40)	0.60 (0.59-0.77)	1.00 (0.99-1.00)	0.55 (95% CI 0.38-0.70)
NPI (inter-eye mean, median in timebin)	0.75 (0.40-0.76)	0.00 (0.00-0.00)	0.00 (0.00-0.40)	0.75 (0.40-0.76)	1.00 (0.99-1.00)	0.51 (95% CI 0.35-0.66)
NPI (inter-eye min, max in timebin)	0.68 (0.42-0.69)	0.00 (0.00-0.00)	0.50 (0.40-0.50)	0.68 (0.42-0.69)	1.00 (0.99-1.00)	0.54 (95% CI 0.41-0.70)
NPI (inter-eye min, min in timebin)	0.77 (0.73-0.81)	0.00 (0.00-0.00)	0.00 (0.00-0.20)	0.77 (0.74-0.81)	1.00 (0.99-1.00)	0.56 (95% CI 0.39-0.71)
NPI (inter-eye min, median in timebin)	0.55 (0.53-0.86)	0.00 (0.00-0.00)	0.00 (0.00-0.40)	0.55 (0.53-0.86)	1.00 (0.99-1.00)	0.52 (95% CI 0.35-0.67)
NPI (inter-eye max, max in timebin)	0.60 (0.43-0.60)	0.00 (0.00-0.00)	0.20 (0.00-0.60)	0.60 (0.42-0.60)	1.00 (0.99-1.00)	0.57 (95% CI 0.45-0.71)
NPI (inter-eye max, min in timebin)	0.68 (0.68-0.69)	0.00 (0.00-0.00)	0.20 (0.00-0.40)	0.68 (0.68-0.69)	1.00 (0.99-1.00)	0.55 (95% CI 0.38-0.71)
NPI (inter-eye max, median in timebin)	0.70 (0.55-0.79)	0.00 (0.00-0.00)	0.00 (0.00-0.20)	0.70 (0.55-0.80)	1.00 (0.99-1.00)	0.50 (95% CI 0.33-0.65)
NPI (inter-eye delta, max in timebin)	0.65 (0.65-0.66)	0.00 (0.00-0.01)	0.50 (0.50-0.50)	0.65 (0.65-0.66)	1.00 (1.00-1.00)	0.57 (95% CI 0.40-0.72)
NPI (inter-eye delta, min in timebin)	0.38 (0.37-0.39)	0.00 (0.00-0.00)	1.00 (0.40-1.00)	0.38 (0.37-0.39)	1.00 (0.99-1.00)	0.56 (95% CI 0.44-0.70)
NPI (inter-eye delta, median in timebin)	0.63 (0.53-0.80)	0.00 (0.00-0.00)	0.40 (0.00-0.50)	0.63 (0.53-0.80)	1.00 (0.99-1.00)	0.52 (95% CI 0.36-0.69)
CV (inter-eye mean, max in timebin)	0.71 (0.70-0.71)	0.01 (0.00-0.01)	1.00 (0.75-1.00)	0.70 (0.70-0.71)	1.00 (1.00-1.00)	0.78 (95% CI 0.65-0.88)

CV (inter-eye mean, min in timebin)	0.56 (0.56-0.64)	0.01 (0.00-0.01)	0.80 (0.75-1.00)	0.56 (0.56-0.64)	1.00 (1.00-1.00)	0.72 (95% CI 0.62-0.81)
CV (inter-eye mean, median in timebin)	0.66 (0.66-0.67)	0.01 (0.01-0.01)	1.00 (0.80-1.00)	0.66 (0.66-0.67)	1.00 (1.00-1.00)	0.77 (95% CI 0.65-0.86)
CV (inter-eye min, max in timebin)	0.73 (0.67-0.74)	0.01 (0.00-0.01)	1.00 (0.75-1.00)	0.73 (0.67-0.74)	1.00 (1.00-1.00)	0.80 (95% CI 0.67-0.90)
CV (inter-eye min, min in timebin)	0.61 (0.60-0.61)	0.01 (0.00-0.01)	1.00 (0.75-1.00)	0.61 (0.60-0.61)	1.00 (1.00-1.00)	0.76 (95% CI 0.67-0.84)
CV (inter-eye min, median in timebin)	0.70 (0.70-0.72)	0.01 (0.00-0.01)	1.00 (0.75-1.00)	0.70 (0.70-0.72)	1.00 (1.00-1.00)	0.79 (95% CI 0.67-0.88)
CV (inter-eye max, max in timebin)	0.66 (0.66-0.67)	0.01 (0.00-0.01)	0.80 (0.75-1.00)	0.66 (0.66-0.66)	1.00 (1.00-1.00)	0.73 (95% CI 0.59-0.86)
CV (inter-eye max, min in timebin)	0.62 (0.62-0.62)	0.00 (0.00-0.01)	0.50 (0.50-0.75)	0.62 (0.62-0.62)	1.00 (1.00-1.00)	0.65 (95% CI 0.50-0.78)
CV (inter-eye max, median in timebin)	0.57 (0.56-0.71)	0.01 (0.00-0.01)	0.60 (0.50-0.75)	0.57 (0.56-0.71)	1.00 (1.00-1.00)	0.70 (95% CI 0.56-0.83)
CV (inter-eye delta, max in timebin)	0.36 (0.25-0.45)	0.00 (0.00-0.00)	0.00 (0.00-0.50)	0.36 (0.26-0.45)	0.99 (0.99-0.99)	0.51 (95% CI 0.34-0.66)
CV (inter-eye delta, min in timebin)	0.64 (0.60-0.64)	0.00 (0.00-0.01)	0.50 (0.50-0.67)	0.64 (0.60-0.64)	1.00 (1.00-1.00)	0.67 (95% CI 0.51-0.82)
CV (inter-eye delta, median in timebin)	0.64 (0.60-0.71)	0.00 (0.00-0.01)	0.60 (0.50-0.67)	0.64 (0.60-0.72)	1.00 (1.00-1.00)	0.60 (95% CI 0.43-0.76)
**8h timebin**						
NPI (inter-eye mean, max in timebin)	0.53 (0.51-0.54)	0.00 (0.00-0.00)	0.40 (0.20-1.00)	0.53 (0.51-0.54)	1.00 (0.99-1.00)	0.54 (95% CI 0.42-0.66)
NPI (inter-eye mean, min in timebin)	0.64 (0.63-0.64)	0.00 (0.00-0.01)	0.40 (0.00-0.80)	0.64 (0.64-0.64)	1.00 (1.00-1.00)	0.56 (95% CI 0.37-0.72)
NPI (inter-eye mean, median in timebin)	0.46 (0.45-0.71)	0.00 (0.00-0.00)	0.00 (0.00-0.40)	0.46 (0.45-0.71)	1.00 (0.99-1.00)	0.53 (95% CI 0.37-0.67)
NPI (inter-eye min, max in timebin)	0.46 (0.46-0.47)	0.00 (0.00-0.00)	1.00 (0.60-1.00)	0.46 (0.46-0.47)	1.00 (1.00-1.00)	0.51 (95% CI 0.38-0.65)

NPI (inter-eye min, min in timebin)	0.78 (0.77-0.83)	0.00 (0.00-0.00)	0.00 (0.00-0.40)	0.78 (0.77-0.83)	1.00 (1.00-1.00)	0.56 (95% CI 0.37-0.72)
NPI (inter-eye min, median in timebin)	0.55 (0.54-0.70)	0.00 (0.00-0.00)	0.00 (0.00-0.40)	0.55 (0.54-0.71)	1.00 (0.99-1.00)	0.53 (95% CI 0.37-0.68)
NPI (inter-eye max, max in timebin)	0.47 (0.21-0.58)	0.00 (0.00-0.00)	0.60 (0.20-1.00)	0.47 (0.21-0.58)	1.00 (1.00-1.00)	0.56 (95% CI 0.45-0.69)
NPI (inter-eye max, min in timebin)	0.72 (0.72-0.73)	0.00 (0.00-0.00)	0.20 (0.00-0.40)	0.73 (0.72-0.73)	1.00 (1.00-1.00)	0.56 (95% CI 0.38-0.73)
NPI (inter-eye max, median in timebin)	0.48 (0.47-0.68)	0.00 (0.00-0.00)	0.00 (0.00-0.20)	0.49 (0.47-0.68)	1.00 (0.99-1.00)	0.53 (95% CI 0.37-0.67)
NPI (inter-eye delta, max in timebin)	0.70 (0.70-0.77)	0.01 (0.00-0.01)	0.50 (0.40-0.50)	0.70 (0.70-0.77)	1.00 (1.00-1.00)	0.59 (95% CI 0.45-0.72)
NPI (inter-eye delta, min in timebin)	0.44 (0.43-0.46)	0.00 (0.00-0.00)	0.50 (0.40-0.50)	0.44 (0.43-0.46)	1.00 (0.99-1.00)	0.57 (95% CI 0.45-0.70)
NPI (inter-eye delta, median in timebin)	0.70 (0.63-0.76)	0.00 (0.00-0.00)	0.40 (0.00-0.50)	0.70 (0.63-0.77)	1.00 (0.99-1.00)	0.52 (95% CI 0.38-0.67)
CV (inter-eye mean, max in timebin)	0.67 (0.66-0.67)	0.01 (0.00-0.01)	1.00 (0.75-1.00)	0.67 (0.66-0.67)	1.00 (1.00-1.00)	0.79 (95% CI 0.66-0.89)
CV (inter-eye mean, min in timebin)	0.60 (0.52-0.61)	0.00 (0.00-0.01)	0.80 (0.75-1.00)	0.60 (0.52-0.61)	1.00 (1.00-1.00)	0.71 (95% CI 0.60-0.81)
CV (inter-eye mean, median in timebin)	0.64 (0.64-0.66)	0.01 (0.00-0.01)	1.00 (0.75-1.00)	0.64 (0.64-0.65)	1.00 (1.00-1.00)	0.76 (95% CI 0.65-0.86)
CV (inter-eye min, max in timebin)	0.70 (0.69-0.70)	0.01 (0.00-0.01)	1.00 (0.75-1.00)	0.70 (0.69-0.70)	1.00 (1.00-1.00)	0.82 (95% CI 0.69-0.91)
CV (inter-eye min, min in timebin)	0.57 (0.56-0.61)	0.00 (0.00-0.01)	0.75 (0.50-1.00)	0.56 (0.56-0.61)	1.00 (1.00-1.00)	0.75 (95% CI 0.65-0.85)
CV (inter-eye min, median in timebin)	0.71 (0.71-0.71)	0.01 (0.00-0.01)	1.00 (0.75-1.00)	0.71 (0.71-0.71)	1.00 (1.00-1.00)	0.80 (95% CI 0.67-0.89)
CV (inter-eye max, max in timebin)	0.70 (0.69-0.71)	0.00 (0.00-0.01)	0.75 (0.50-0.80)	0.70 (0.69-0.71)	1.00 (1.00-1.00)	0.73 (95% CI 0.59-0.85)
CV (inter-eye max, min in timebin)	0.60 (0.59-0.60)	0.00 (0.00-0.01)	0.50 (0.50-0.75)	0.60 (0.59-0.60)	1.00 (1.00-1.00)	0.63 (95% CI 0.48-0.78)

CV (inter-eye max, median in timebin)	0.71 (0.52-0.72)	0.00 (0.00-0.01)	0.50 (0.50-0.60)	0.71 (0.52-0.72)	1.00 (1.00-1.00)	0.69 (95% CI 0.55-0.82)
CV (inter-eye delta, max in timebin)	0.42 (0.41-0.69)	0.00 (0.00-0.00)	0.50 (0.00-0.50)	0.42 (0.41-0.69)	1.00 (0.99-1.00)	0.55 (95% CI 0.40-0.71)
CV (inter-eye delta, min in timebin)	0.67 (0.66-0.72)	0.00 (0.00-0.01)	0.50 (0.50-0.67)	0.67 (0.66-0.72)	1.00 (1.00-1.00)	0.69 (95% CI 0.52-0.85)
CV (inter-eye delta, median in timebin)	0.72 (0.72-0.74)	0.00 (0.00-0.01)	0.50 (0.40-0.50)	0.72 (0.72-0.73)	1.00 (1.00-1.00)	0.62 (95% CI 0.44-0.80)
12h timebin						
NPI (inter-eye mean, max in timebin)	0.26 (0.13-0.33)	0.00 (0.00-0.00)	0.67 (0.40-1.00)	0.25 (0.13-0.32)	1.00 (0.99-1.00)	0.50 (95% CI 0.39-0.62)
NPI (inter-eye mean, min in timebin)	0.68 (0.68-0.72)	0.01 (0.00-0.01)	0.50 (0.40-0.80)	0.68 (0.68-0.72)	1.00 (1.00-1.00)	0.63 (95% CI 0.47-0.77)
NPI (inter-eye mean, median in timebin)	0.42 (0.40-0.45)	0.01 (0.00-0.01)	0.80 (0.67-1.00)	0.42 (0.40-0.45)	1.00 (1.00-1.00)	0.59 (95% CI 0.46-0.72)
NPI (inter-eye min, max in timebin)	0.30 (0.29-0.45)	0.00 (0.00-0.00)	0.40 (0.33-1.00)	0.29 (0.29-0.45)	0.99 (0.99-1.00)	0.53 (95% CI 0.40-0.64)
NPI (inter-eye min, min in timebin)	0.77 (0.75-0.81)	0.01 (0.00-0.01)	0.40 (0.33-0.60)	0.77 (0.75-0.82)	1.00 (1.00-1.00)	0.63 (95% CI 0.47-0.78)
NPI (inter-eye min, median in timebin)	0.63 (0.53-0.84)	0.01 (0.00-0.01)	0.33 (0.25-0.60)	0.63 (0.53-0.84)	1.00 (1.00-1.00)	0.61 (95% CI 0.48-0.74)
NPI (inter-eye max, max in timebin)	0.17 (0.15-0.53)	0.00 (0.00-0.01)	1.00 (0.40-1.00)	0.16 (0.15-0.53)	1.00 (0.99-1.00)	0.53 (95% CI 0.42-0.64)
NPI (inter-eye max, min in timebin)	0.71 (0.66-0.73)	0.00 (0.00-0.01)	0.40 (0.33-0.80)	0.71 (0.66-0.73)	1.00 (1.00-1.00)	0.61 (95% CI 0.45-0.75)
NPI (inter-eye max, median in timebin)	0.46 (0.41-0.46)	0.00 (0.00-0.01)	0.80 (0.40-1.00)	0.46 (0.41-0.46)	1.00 (1.00-1.00)	0.58 (95% CI 0.44-0.71)
NPI (inter-eye delta, max in timebin)	0.42 (0.40-0.74)	0.00 (0.00-0.01)	0.60 (0.33-0.80)	0.42 (0.40-0.74)	1.00 (1.00-1.00)	0.61 (95% CI 0.48-0.74)
NPI (inter-eye delta, min in timebin)	0.48 (0.48-0.48)	0.00 (0.00-0.00)	0.40 (0.00-0.67)	0.48 (0.47-0.48)	1.00 (0.99-1.00)	0.53 (95% CI 0.41-0.63)

NPI (inter-eye delta, median in timebin)	0.67 (0.66-0.68)	0.01 (0.01-0.01)	0.60 (0.40-0.67)	0.67 (0.66-0.68)	1.00 (1.00-1.00)	0.58 (95% CI 0.41-0.71)
CV (inter-eye mean, max in timebin)	0.70 (0.70-0.71)	0.01 (0.01-0.01)	0.75 (0.67-1.00)	0.70 (0.70-0.71)	1.00 (1.00-1.00)	0.71 (95% CI 0.55-0.83)
CV (inter-eye mean, min in timebin)	0.52 (0.51-0.54)	0.01 (0.00-0.01)	0.67 (0.60-1.00)	0.52 (0.51-0.54)	1.00 (1.00-1.00)	0.59 (95% CI 0.45-0.72)
CV (inter-eye mean, median in timebin)	0.66 (0.66-0.67)	0.01 (0.00-0.01)	0.60 (0.33-0.75)	0.66 (0.66-0.67)	1.00 (1.00-1.00)	0.67 (95% CI 0.51-0.80)
CV (inter-eye min, max in timebin)	0.74 (0.74-0.74)	0.01 (0.01-0.02)	0.67 (0.60-0.80)	0.74 (0.74-0.74)	1.00 (1.00-1.00)	0.72 (95% CI 0.56-0.85)
CV (inter-eye min, min in timebin)	0.67 (0.66-0.67)	0.00 (0.00-0.01)	0.60 (0.33-0.60)	0.67 (0.66-0.67)	1.00 (1.00-1.00)	0.63 (95% CI 0.48-0.76)
CV (inter-eye min, median in timebin)	0.64 (0.64-0.70)	0.01 (0.01-0.01)	0.60 (0.50-0.67)	0.64 (0.64-0.70)	1.00 (1.00-1.00)	0.69 (95% CI 0.53-0.83)
CV (inter-eye max, max in timebin)	0.76 (0.76-0.77)	0.01 (0.00-0.01)	0.50 (0.33-0.60)	0.76 (0.76-0.77)	1.00 (1.00-1.00)	0.66 (95% CI 0.51-0.79)
CV (inter-eye max, min in timebin)	0.54 (0.53-0.55)	0.01 (0.00-0.01)	0.50 (0.33-0.60)	0.54 (0.53-0.55)	1.00 (1.00-1.00)	0.54 (95% CI 0.39-0.69)
CV (inter-eye max, median in timebin)	0.77 (0.76-0.78)	0.01 (0.01-0.01)	0.50 (0.33-0.60)	0.77 (0.76-0.78)	1.00 (1.00-1.00)	0.61 (95% CI 0.46-0.75)
CV (inter-eye delta, max in timebin)	0.35 (0.33-0.66)	0.00 (0.00-0.00)	0.33 (0.25-1.00)	0.34 (0.33-0.66)	0.99 (0.99-1.00)	0.55 (95% CI 0.41-0.68)
CV (inter-eye delta, min in timebin)	0.73 (0.59-0.73)	0.01 (0.01-0.01)	0.67 (0.40-0.67)	0.73 (0.59-0.73)	1.00 (1.00-1.00)	0.70 (95% CI 0.56-0.83)
CV (inter-eye delta, median in timebin)	0.62 (0.61-0.63)	0.01 (0.01-0.01)	0.67 (0.67-0.75)	0.62 (0.61-0.63)	1.00 (1.00-1.00)	0.68 (95% CI 0.54-0.81)
**24h timebin**						
NPI (inter-eye mean, max in timebin)	0.27 (0.16-0.31)	0.00 (0.00-0.01)	0.80 (0.75-1.00)	0.26 (0.16-0.31)	1.00 (1.00-1.00)	0.55 (95% CI 0.45-0.65)
NPI (inter-eye mean, min in timebin)	0.71 (0.71-0.72)	0.01 (0.01-0.01)	0.50 (0.40-0.80)	0.71 (0.71-0.72)	1.00 (1.00-1.00)	0.65 (95% CI 0.50-0.77)

NPI (inter-eye mean, median in timebin)	0.39 (0.38-0.40)	0.01 (0.00-0.01)	0.80 (0.75-0.80)	0.39 (0.38-0.40)	1.00 (1.00-1.00)	0.58 (95% CI 0.46-0.68)
NPI (inter-eye min, max in timebin)	0.12 (0.10-0.15)	0.01 (0.01-0.01)	1.00 (1.00-1.00)	0.11 (0.10-0.15)	1.00 (1.00-1.00)	0.50 (95% CI 0.41-0.61)
NPI (inter-eye min, min in timebin)	0.74 (0.74-0.75)	0.01 (0.01-0.01)	0.50 (0.40-0.60)	0.74 (0.74-0.75)	1.00 (1.00-1.00)	0.64 (95% CI 0.49-0.76)
NPI (inter-eye min, median in timebin)	0.40 (0.40-0.41)	0.01 (0.00-0.01)	0.80 (0.50-0.80)	0.40 (0.40-0.41)	1.00 (0.99-1.00)	0.59 (95% CI 0.48-0.69)
NPI (inter-eye max, max in timebin)	0.67 (0.20-0.69)	0.01 (0.00-0.01)	0.50 (0.40-0.80)	0.67 (0.20-0.69)	1.00 (0.99-1.00)	0.59 (95% CI 0.49-0.70)
NPI (inter-eye max, min in timebin)	0.72 (0.72-0.73)	0.01 (0.01-0.01)	0.50 (0.40-0.80)	0.72 (0.72-0.73)	1.00 (1.00-1.00)	0.65 (95% CI 0.51-0.77)
NPI (inter-eye max, median in timebin)	0.40 (0.36-0.41)	0.01 (0.00-0.01)	0.80 (0.75-0.80)	0.39 (0.36-0.41)	1.00 (1.00-1.00)	0.55 (95% CI 0.44-0.65)
NPI (inter-eye delta, max in timebin)	0.62 (0.62-0.76)	0.01 (0.01-0.01)	0.60 (0.50-0.60)	0.62 (0.62-0.76)	1.00 (1.00-1.00)	0.67 (95% CI 0.54-0.79)
NPI (inter-eye delta, min in timebin)	0.12 (0.10-0.30)	0.00 (0.00-0.01)	0.75 (0.50-1.00)	0.12 (0.10-0.29)	1.00 (0.99-1.00)	0.52 (95% CI 0.42-0.63)
NPI (inter-eye delta, median in timebin)	0.43 (0.38-0.50)	0.01 (0.01-0.01)	0.80 (0.75-0.80)	0.43 (0.38-0.50)	1.00 (1.00-1.00)	0.63 (95% CI 0.52-0.73)
CV (inter-eye mean, max in timebin)	0.70 (0.70-0.71)	0.01 (0.01-0.02)	0.75 (0.50-0.80)	0.70 (0.70-0.71)	1.00 (1.00-1.00)	0.71 (95% CI 0.58-0.83)
CV (inter-eye mean, min in timebin)	0.75 (0.73-0.75)	0.00 (0.00-0.00)	0.00 (0.00-0.20)	0.75 (0.73-0.76)	0.99 (0.99-1.00)	0.55 (95% CI 0.42-0.69)
CV (inter-eye mean, median in timebin)	0.78 (0.77-0.78)	0.01 (0.01-0.01)	0.40 (0.25-0.60)	0.78 (0.77-0.79)	1.00 (1.00-1.00)	0.64 (95% CI 0.51-0.78)
CV (inter-eye min, max in timebin)	0.71 (0.70-0.73)	0.01 (0.01-0.02)	0.80 (0.75-1.00)	0.71 (0.70-0.73)	1.00 (1.00-1.00)	0.72 (95% CI 0.58-0.84)
CV (inter-eye min, min in timebin)	0.76 (0.68-0.77)	0.00 (0.00-0.01)	0.20 (0.00-0.40)	0.76 (0.68-0.78)	1.00 (0.99-1.00)	0.57 (95% CI 0.44-0.71)
CV (inter-eye min, median in timebin)	0.78 (0.57-0.78)	0.01 (0.01-0.01)	0.40 (0.25-0.60)	0.78 (0.57-0.78)	1.00 (1.00-1.00)	0.67 (95% CI 0.54-0.80)

CV (inter-eye max, max in timebin)	0.67 (0.65-0.68)	0.01 (0.01-0.01)	0.50 (0.40-0.60)	0.66 (0.65-0.68)	1.00 (1.00-1.00)	0.64 (95% CI 0.49-0.78)
CV (inter-eye max, min in timebin)	0.82 (0.81-0.86)	0.00 (0.00-0.00)	0.00 (0.00-0.00)	0.83 (0.81-0.86)	0.99 (0.99-1.00)	0.52 (95% CI 0.38-0.67)
CV (inter-eye max, median in timebin)	0.73 (0.72-0.78)	0.01 (0.00-0.01)	0.25 (0.00-0.60)	0.73 (0.72-0.78)	1.00 (1.00-1.00)	0.59 (95% CI 0.46-0.74)
CV (inter-eye delta, max in timebin)	0.64 (0.36-0.67)	0.00 (0.00-0.00)	0.40 (0.20-0.50)	0.64 (0.35-0.67)	0.99 (0.99-0.99)	0.50 (95% CI 0.36-0.64)
CV (inter-eye delta, min in timebin)	0.49 (0.46-0.81)	0.01 (0.01-0.01)	0.50 (0.50-0.60)	0.49 (0.46-0.81)	1.00 (0.99-1.00)	0.61 (95% CI 0.48-0.74)
CV (inter-eye delta, median in timebin)	0.84 (0.84-0.84)	0.01 (0.01-0.02)	0.50 (0.25-0.50)	0.84 (0.84-0.85)	1.00 (1.00-1.00)	0.61 (95% CI 0.46-0.76)

Legend: ROC AUC was computed over the entire cohort. 95% confidence intervals were obtained via bootstrapping. Metrics of binary discrimination were obtained through 5-fold cross-validation and reported as median (IQR). CV: constriction velocity; NPi: Neurological pupil index; DCI: delayed cerebral ischemia; CI: confidence interval; IQR: interquartile range; ROC AUC: area under receiver operating characteristic curve; PPV: positive predictive value; NPV: negative predictive value.

Supplemental Results 1.
Performance of inter-eye delta NPi for the prediction of DCI.

An inter-eye delta of NPi > 0.7 is associated with worse discharge modified Rankin scores in patients with subarachnoid haemorrhage from the END-PANIC registry (1). Consequently, the maximum inter-eye delta NPi in timebins from 6h to 24h was evaluated separately with an *a priori* defined threshold of 0.7. Overall, this feature achieved poor predictive performance for the detection of delayed cerebral ischemia due to low sensitivity (6-9%).

Timebin size	Accuracy	Precision (PPV)	Recall (Sensitivity)	Specificity	NPV
6h	0.87	0.00	0.06	0.87	0.99
8h	0.85	0.00	0.06	0.86	0.99
12h	0.82	0.00	0.06	0.83	0.99
24h	0.77	0.00	0.09	0.78	0.99

Legend: Performance of inter-eye delta NPi (maximum in timebin) for the prediction of DCI. No normalisation was applied. An *a priori* defined threshold of 0.7 was used. NPi: Neurological pupil index; DCI: delayed cerebral ischemia; PPV: positive predictive value; NPV: negative predictive value.

Supplemental Results 2.

Coefficients for ordinal logistic regression analysis.

A. Univariable association of CV and 1-year modified Rankin scales

Independent variable	Coefficient	Standard error	Test statistic (z)	95% CI
CV	-1.84	0.43	-4.28	[-2.68; -1.00]

Legend: Coefficients for an ordinal logistic regression model with 1-year mRs as dependent variable and CV as independent variable. For every patient the median of inter-eye minimum CV over the whole ICU-stay was used. CV: constriction velocity; mRs: modified Rankin scale; CI: confidence interval.

B. Univariable association of NPi and 1-year modified Rankin scales

Independent variable	Coefficient	Standard error	Test statistic (z)	95% CI
NPi	-0.93	0.26	-3.56	[-1.44; -0.42]

Legend: Coefficients for an ordinal logistic regression model with 1-year mRs as dependent variable and NPi as independent variable. For every patient the median of inter-eye minimum NPi over the whole ICU-stay was used. NPi: Neurological pupil index; mRs: modified Rankin scale; CI: confidence interval.

C. Multivariable association of pupillometry features and 1-year modified Rankin scales

Independent variable	Coefficient	Standard error	Test statistic (z)	95% CI
CV	-1.35	0.45	-2.99	[-2.23; -0.47]
NPi	-0.09	0.30	-0.31	[-0.67; 0.49]
Age	0.02	0.02	1.01	[-0.02; 0.06]
WFNS	0.53	0.19	2.75	[0.15; 0.91]
Fisher	0.67	0.76	0.89	[-0.81; 2.16]

Legend: Coefficients for an ordinal logistic regression model with 1-year mRs as dependent variable and CV, NPi as well as age, WFNS and modified Fisher scales independent variables. For every patient the median of inter-eye minimum NPi and CV over the whole ICU-stay was used. CV: constriction velocity; NPi: Neurological pupil index; mRs: modified Rankin scale; CI: confidence interval; WFNS: World federation of neurological surgeons.

Supplemental Figure 6.

Sensitivity analysis: study flow chart.

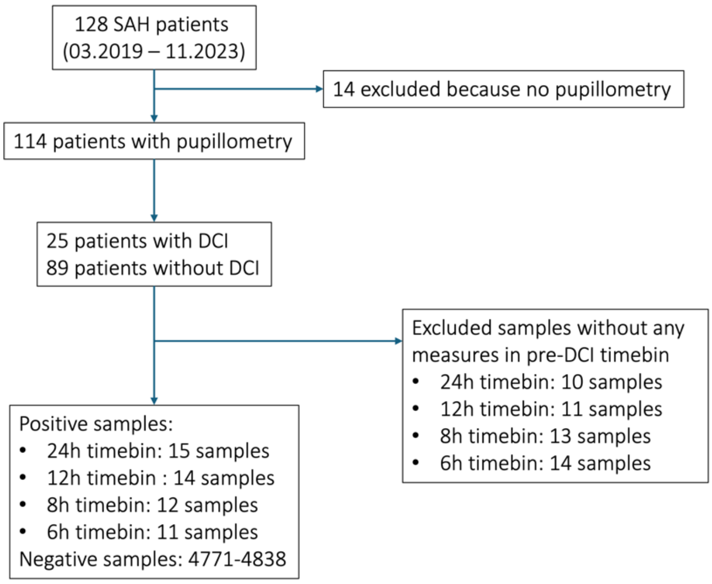

Legend: Study flow chart of the sensitivity analysis in which the evidence of focal infarction not related to aneurysm occlusion and not attributed to other causes was required for the definition of DCI. SAH: subarachnoid haemorrhage; DCI: delayed cerebral ischemia.

Supplemental Figure 7.

Sensitivity analysis: Boxplots of non-normalized CV across timebins according to the development of DCI with infarction.

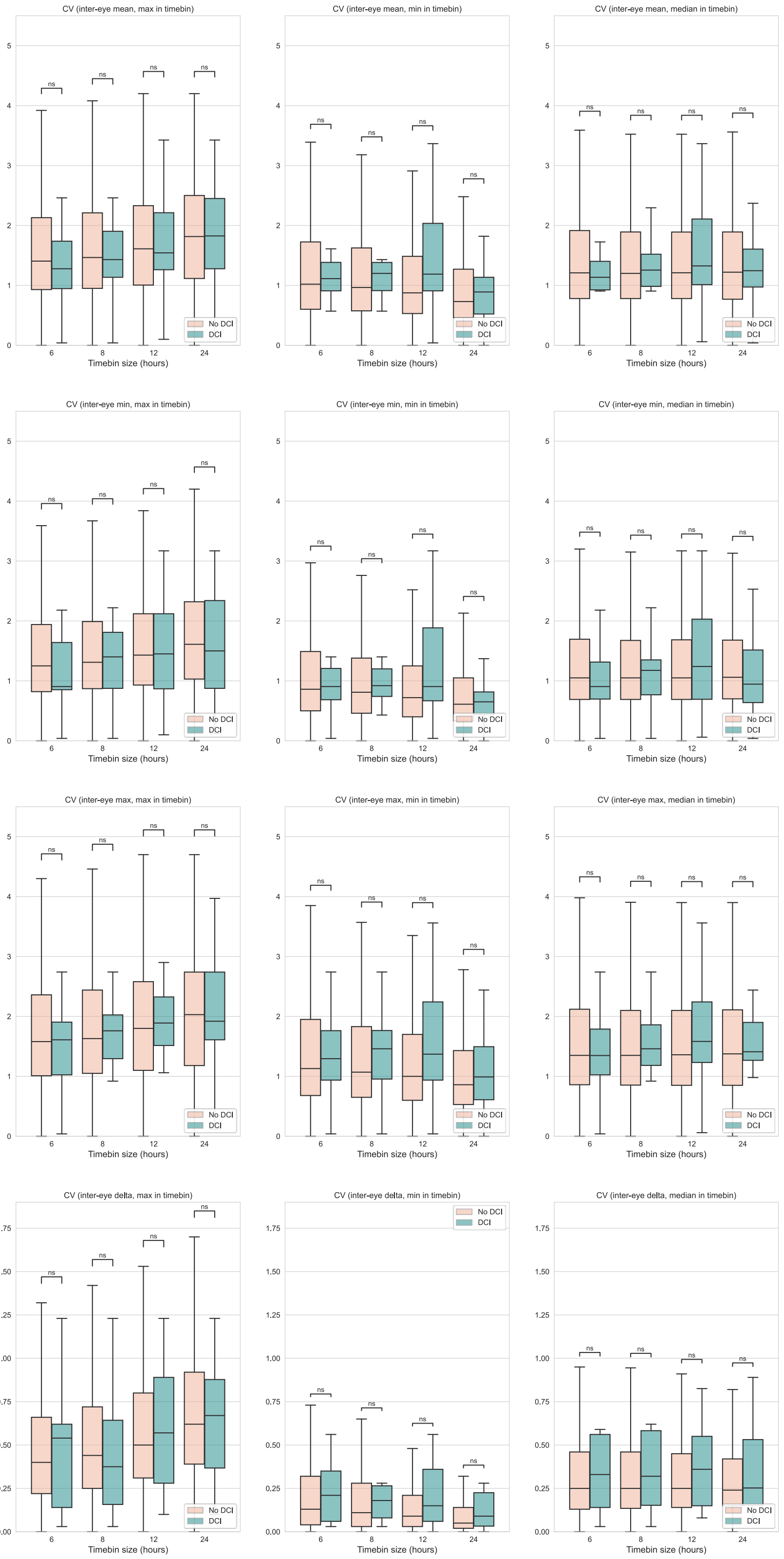

__Legend__: Samples with DCI are color-coded in green, negative samples in salmon. Non-normalised CV is expressed in $mm.s^{-1}$. CV: constriction velocity; DCI: delayed cerebral ischemia; ns: non-significant; *: p-value < 0.05, **: p-value < 0.01; ***: p-value < 0.001; ****: p-value < 0.0001.

Supplemental Figure 8.

Sensitivity analysis: Boxplots of non-normalized NPi across timebins according to the development of DCI with infarction.

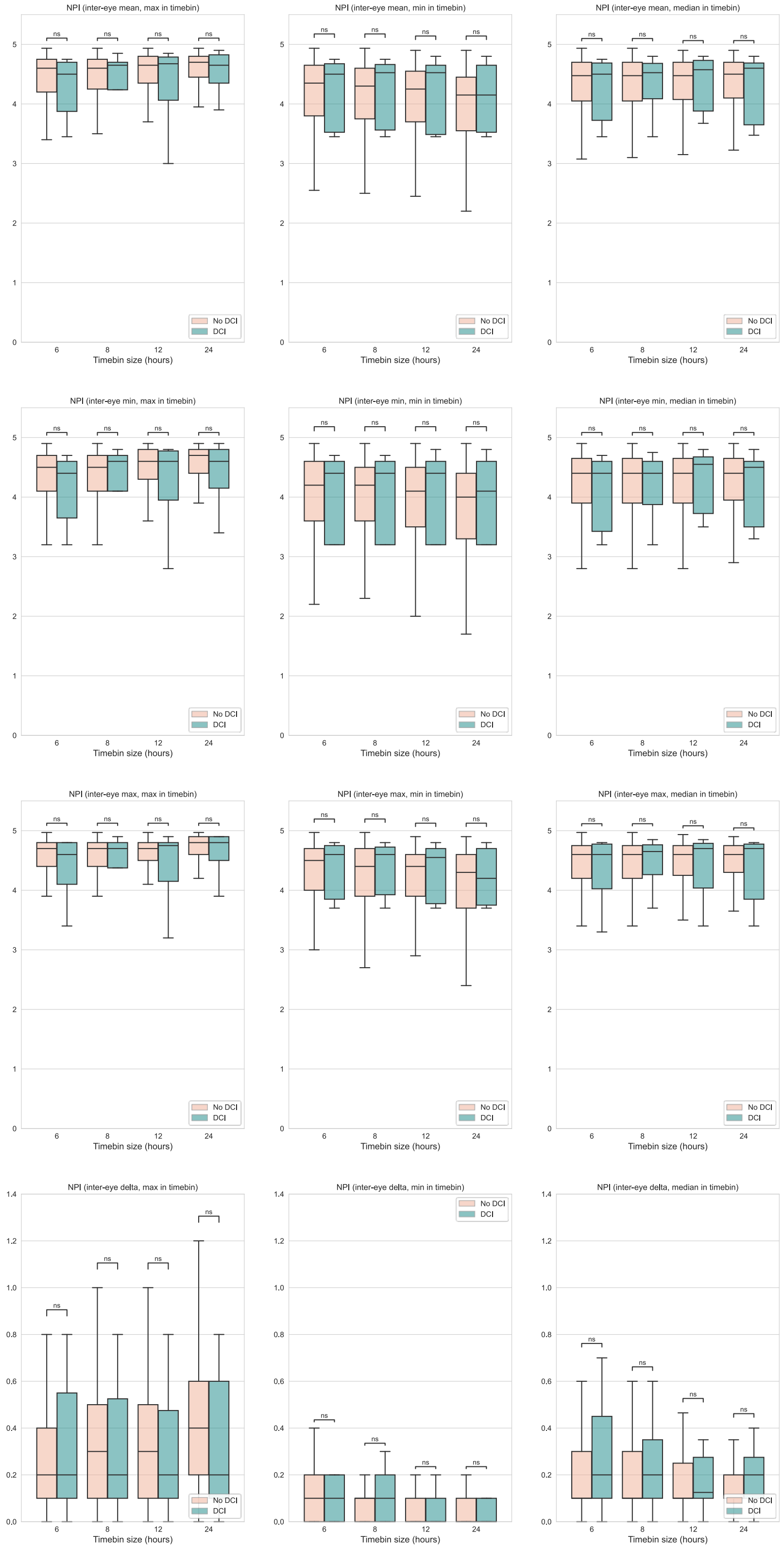

<u>Legend</u>: Samples with DCI are color-coded in green, negative samples in salmon. NPi: Neurological pupil index; DCI: delayed cerebral ischemia; ns: non-significant; *: p-value < 0.05, **: p-value < 0.01; ***: p-value < 0.001; ****: p-value < 0.0001.

Supplemental Figure 9.

Sensitivity analysis: Boxplots of normalized CV across timebins according to the development of DCI with infarction.

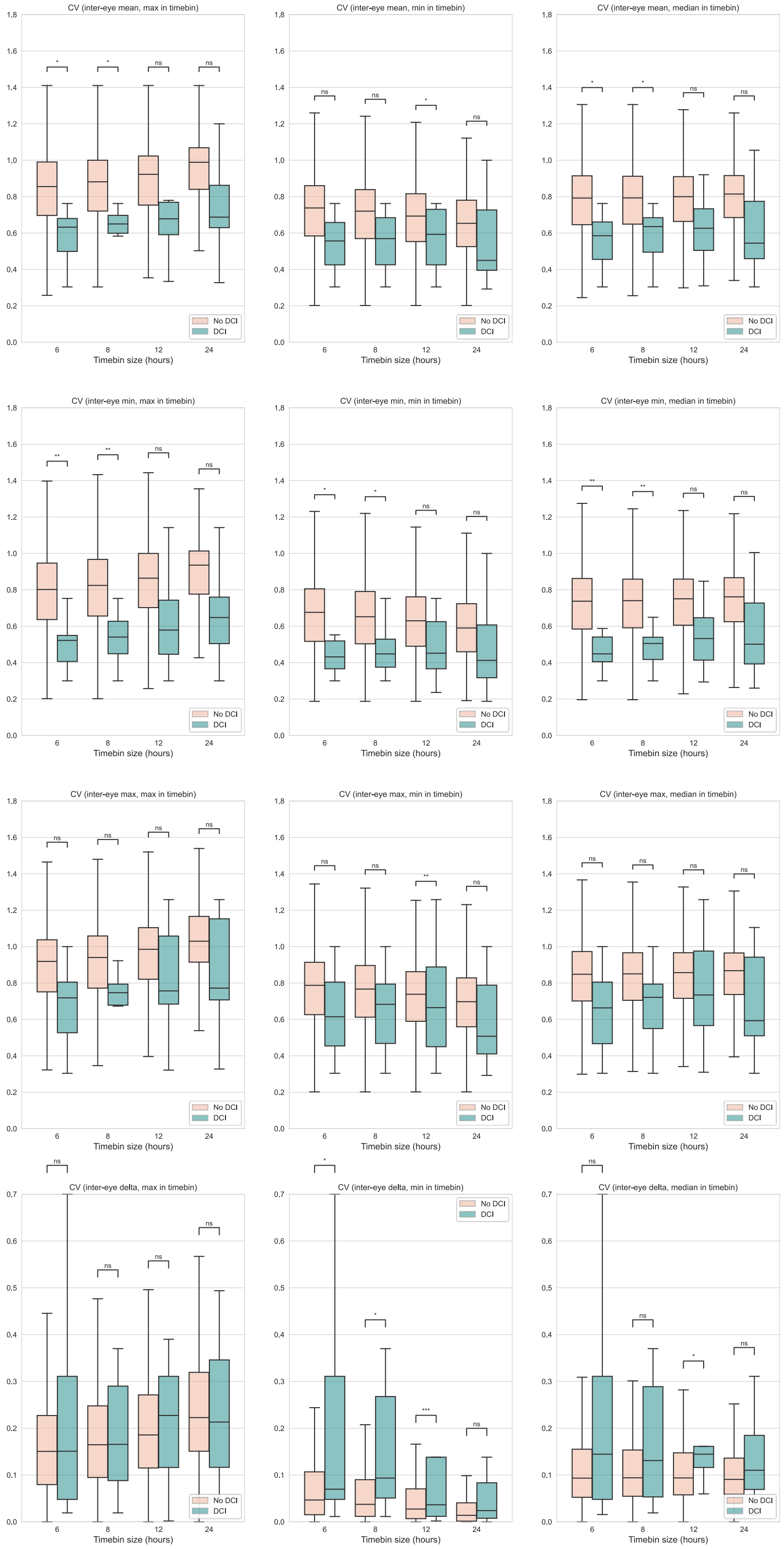

Legend: Samples with DCI are color-coded in green, negative samples in salmon. CV: constriction velocity; DCI: delayed cerebral ischemia; ns: non-significant; *: p-value < 0.05, **: p-value < 0.01; ***: p-value < 0.001; ****: p-value < 0.0001.

Supplemental Figure 10.

Sensitivity analysis: Boxplots of normalized NPi across timebins according to the development of DCI with infarction.

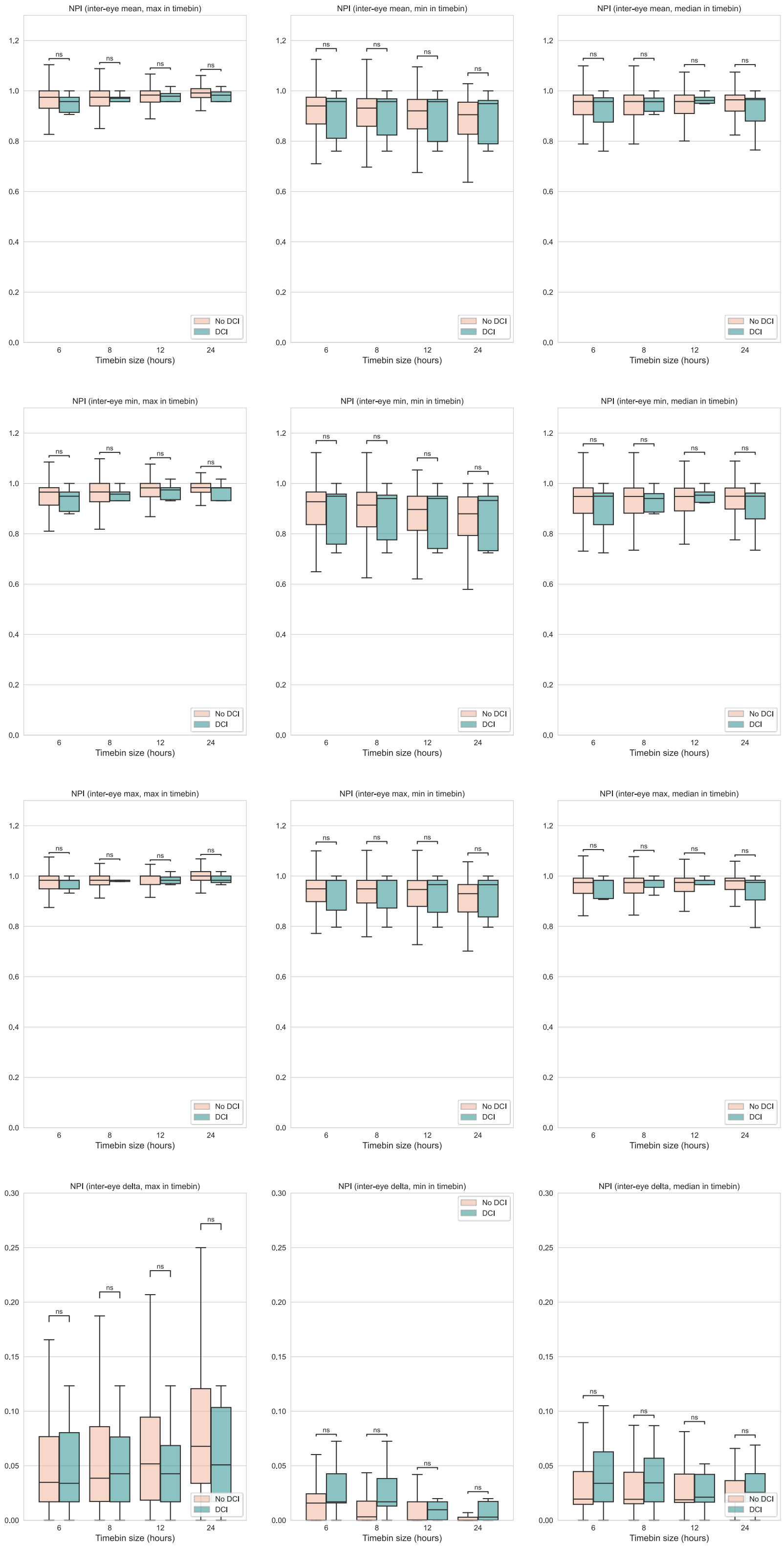

Legend: Samples with DCI are color-coded in green, negative samples in salmon. NPi: Neurological pupil index; DCI: delayed cerebral ischemia; ns: non-significant; *: p-value < 0.05, **: p-value < 0.01; ***: p-value < 0.001; ****: p-value < 0.0001.

Supplemental Methods 3.

Normalized threshold as a function of relative ratio.

The normalized metric threshold (T_{norm}) can be expressed as:

$$T_{norm} > \frac{m_t + 1}{\max\limits_{0 \le i < t} m_i + 1}$$

equivalent to:

$$T_{norm} * \left(\max_{0 \le i < t} m_i + 1\right) - 1 > m_t$$

To express as a relative ratio, we divide by $\max\limits_{0 \le i < t} m_i$:

$$\frac{T_{norm} * \left(\max\limits_{0 \le i < t} m_i + 1\right) - 1}{\max\limits_{0 \le i < t} m_i} > \frac{m_t}{\max\limits_{0 \le i < t} m_i}$$

equivalent to:

$$T_{norm} + \frac{(T_{norm} - 1)}{\max\limits_{0 \le i < t} m_i} > \frac{m_t}{\max\limits_{0 \le i < t} m_i}$$

Expressed as a relative reduction in metric:

$$1 - (T_{norm} + \frac{(T_{norm} - 1)}{\max\limits_{0 \le i < t} m_i}) < 1 - \frac{m_t}{\max\limits_{0 \le i < t} m_i}$$

where T_{norm} is the threshold for the normalized metric, m_t is the metric at timepoint t with $m_t > 0$ and $\max\limits_{0 \le i < t} m_i$ the maximum up to timepoint t.

For a given threshold of constriction velocity $T_{norm}(CV)$ of 0.71, this would result in:

$$0.71 - \frac{0.29}{\max\limits_{0 \le i < t} CV_i} > \frac{CV_t}{\max\limits_{0 \le i < t} CV_i}$$

and a relative reduction threshold of:

$$0.29 + \frac{0.29}{\max\limits_{0 \le i < t} CV_i} < 1 - \frac{CV_t}{\max\limits_{0 \le i < t} CV_i}$$

References

1. Privitera CM, Neerukonda SV, Aiyagari V, Yokobori S, Puccio AM, Schneider NJ, et al. A differential of the left eye and right eye neurological pupil index is associated with discharge modified Rankin scores in neurologically injured patients. BMC Neurol. 2022 Jul 22;22(1):273.
